# From Nano to Micro Polyion Complex Vesicles: Synthetic Cells with Membrane-Embedded Enzymes

**DOI:** 10.1021/acsami.5c11988

**Published:** 2025-08-11

**Authors:** Celia Jimenez-Lopez, Roi Lopez-Blanco, Iria Esperon-Abril, Eduardo Fernandez-Megia

**Affiliations:** Centro Singular de Investigación en Química Biolóxica e Materiais Moleculares (CIQUS), Departamento de Química Orgánica, 16780 Universidade de Santiago de Compostela, Jenaro de la Fuente s/n, Santiago de Compostela 15782, Spain

**Keywords:** dendrimer, polyion complex vesicle, PICsome, synthetic cell, hierarchical transfer

## Abstract

Synthetic cells are emerging as cornerstone in our understanding of prebiotic forms of early life and the development of therapeutics. Although several types of vesicles have been proposed for this purpose, their development is often hampered by limited membrane permeability. On the other hand, polyion complex vesicles (PICsomes) with a high permeability for small molecules suffer from a small size (typically sub-200 nm) and low encapsulation efficiency of enzymes (less than 4%). Herein, we describe the peripheral charge density of dendrimers and the ionic strength of the medium as powerful tools in the size tuning of PICsomes via a dendrimer-to-PIC hierarchical transfer of structural information. PICsomes beyond the micron range were readily obtained from a single dendrimer generation (G) and their ability to emulate life-like technologies explored through chemical communication. As opposed to the low protein encapsulation in the lumen of classical PICsomes, a selective enzyme embedding in the PIC membrane was revealed with efficiencies up to 85%. Notably, membrane-embedded enzymes retain high catalytic activity (85% relative to free enzymes), even in the presence of proteases, enabling fast enzymatic cascades between synthetic cell populations.

## Graphical abstract

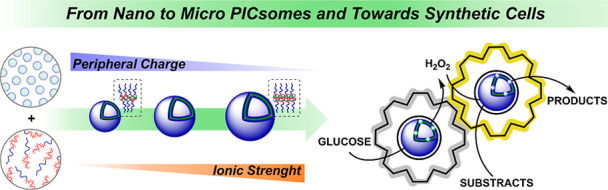

## Introduction

The phenomenon of compartmentalization observed in eukaryotic cells has played a decisive role in the genesis of primitive cells.^1,2^ Although preparing completely functional cells is beyond the reach of current technologies, the de novo construction of synthetic cells that perform natural cellular functions represents a first step toward this goal.^3–7^ In addition, as compartments get better at mimicking natural, living cells, some of the mysteries of biology will be revealed, leading to a deeper understanding of prebiotic forms of early life, as well as the development of synthetic cells with life-like technologies and new therapeutic applications.^8–10^

Among the vesicular systems proposed so far, the self-assembly of lipids and fatty acids has emerged as the dominant paradigm for the origin of life, able to replicate typical cellular properties.^11^ However, lipid membranes suffer from low permeability for small molecules, which prevents continuous activity due to mass transfer. The marked instability of fatty acid vesicles to pH, ionic strength, and multivalent cations also represents significant limitations.^12^ Although alternative vesicles have been proposed by self-assembly of more sophisticated amphiphiles,^10,12^ such as colloidal nanoparticles (colloidosomes), protein–polymer conjugates (proteinosomes), and block copolymers (nanometer-sized polymersomes and micrometer-sized polymer giant vesicles),^13–15^ their preparation often involves the use of organic solvents that might impact encapsulated biomacromolecules. Furthermore, colloidosomes and proteinosomes are subject to technologically complex processes for routine application. As for polymersomes, their low permeability to small molecules and low encapsulation efficiency of biomacromolecules have stimulated the development of more permeable membranes^16–18^ and the use of microfluidics for micron-scale polymer giant vesicles with almost complete enzyme encapsulation efficiencies.^19^ These efforts have enabled the encapsulation of nanosized vesicles within microsized ones, creating compartment-in-compartment architectures that mimic the complex intracellular scenario.^13,20–23^ Alternative innovative approaches to developing sophisticated synthetic cells include the creation of fully protein-based vesicles^24^ and the integration of cell-free protein synthesis mechanisms into vesicles to achieve complex cytomimetic functions.^25^ Also, recent bottom-up approaches have enabled the hierarchical organization of vesicles to gain a deeper insight into the intricacies of intercellular communication and the interactions that occur within natural tissues and organs.^26–28^

Just as the enclosing membrane has determined the evolution of natural cells, modulating membrane properties is essential for developing synthetic cells for therapeutic applications. In the search for alternative membranes with increased permeability, we turned to polyion complex vesicles (PICsomes) originally described by Kataoka,^29,30^ which are prepared in fully aqueous media from oppositely charged block copolymers (or a block copolymer and a polyelectrolyte) at stoichiometric charge ratios.^31,32^ The presence of a neutral hydrophilic block in the block copolymer, usually poly­(ethylene glycol) (PEG), provides neutral vesicles with a nanometric-thick membrane composed of a layer of polyelectrolytes shielded from the internal cavity and surrounding media by hydrophilic PEG chains (Figure 1). Because the PIC membrane ensures permeability for small molecules while retaining macromolecules in the vesicle lumen, PICsomes have found application as enzymatic nanoreactors in vitro and in vivo.^33–36^ Nonetheless, their application as synthetic cells has proven challenging due to their small size (typically sub-200 nm) and poor stability to ionic strength (disassembly under physiological conditions). In fact, microsized PICsomes initially reported in the presence of 150 mM NaCl were later more accurately described as coacervate-like products.^37,38^ In addition, a low encapsulation efficiency of enzymes (typically less than 4%) has also hampered their development as synthetic cells.^33,35,39,40^

**Figure 1:**
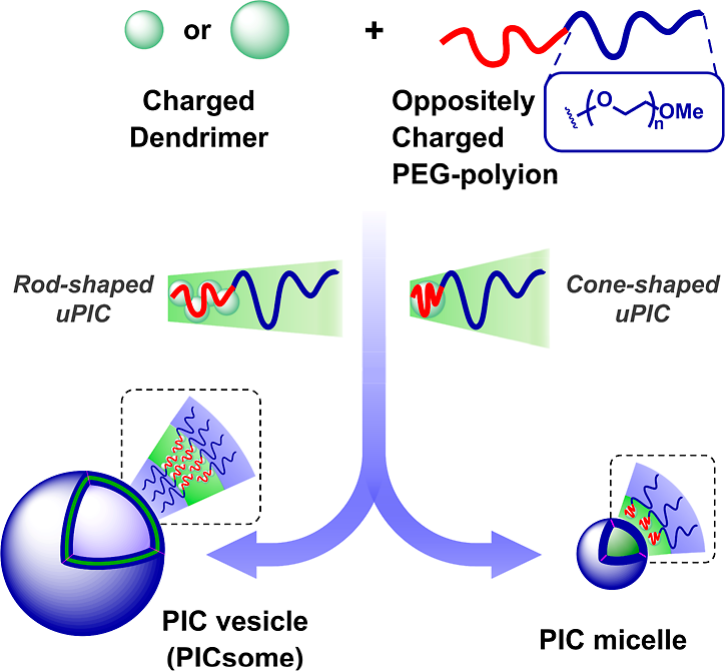
Size and morphology of polyion complex (PIC) micelles and vesicles (PICsomes) depend on the architecture and self-assembly of the monomeric unit PIC (uPIC): cone-shaped uPICs self-assemble into small micelles and rod-shaped uPICs produce larger PICsomes. In turn, the uPIC architecture can be tuned with the G of charged dendrimers (ref 43). While higher G (large and highly charged dendrimers) favor cone-shaped uPICs, lower G (smaller and weakly charged dendrimers) favor rod-shaped uPICs.

The assembly of PICsomes can be described using a simple model such as the packing parameter. It has been proposed that the size and morphology of PIC assemblies (micelles and vesicles) depend on the architecture and self-assembly of the monomeric unit PIC (uPIC)–the minimum neutral assembly formed from oppositely charged species in the early stages of the PIC growth. While cone-shaped uPICs self-assemble into small micelles, rod-shaped uPICs produce larger PICsomes (Figure 1).^41,42^ Inspired by this assembly model, we have described the size tuning of PIC assemblies using charged dendrimers, obtaining PICsomes up to 500 nm^43^ and micelles close to 2 μm.^44^ Dendrimers are polymers composed of repetitive layers of branching units prepared in a controlled iterative fashion, through generations (G) with discrete size and multivalency.^45^ Due to their monodispersity and tree-like globular architecture, they are ideal multivalent templates for the evaluation of new technologies and bioapplications.^46^ Interestingly, tuning the size of PICsomes revealed that reducing the dendrimer G resulted in larger PICsomes.^43^ As dendrimers are recruited into the uPIC to provide charge neutrality (either a small number of large and highly charged dendrimers or a larger number of smaller and weakly charged ones), the increase in size with lower G has been interpreted according to a uPIC progression toward more rod-shaped architectures with better fit into lamellae with lower curvature (Figure 1). Notably, this dendrimer-to-PIC hierarchical transfer of structural information is not attainable by adjusting the molecular weight of linear polymers.^47–50^ Differences in local dynamics between the two types of polymers^51^ explain this dendritic effect,^52,53^ which also results in an unprecedented stability of dendritic PICs toward ionic strength,^43,54–59^ as recently highlighted.^60^

Nevertheless, tuning the size of dendritic PICsomes using the dendrimer G faces certain limitations: (i) the quantized nature of dendrimers restricts access to a single PICsome size per G, and (ii) the largest PICsomes fall short for evaluation as synthetic cells. Therefore, we decided to explore alternative strategies for tailoring the size of PICsomes beyond the micron range using a single dendritic G. This would not only surpass current technological limitations but also result in a significant reduction in the overall synthetic effort. Herein, we describe two complementary strategies to achieve this goal: modulating the peripheral charge density (PCD) of a dendrimer or adjusting the ionic strength of the medium (Figure 2). The resulting PICsomes, with on-demand size control over the micron, have been revealed as interesting synthetic cells with ability to emulate life-like technologies such as efficient compartmentalization, enzyme encapsulation, and chemical communication via enzymatic cascades.

**Figure 2:**
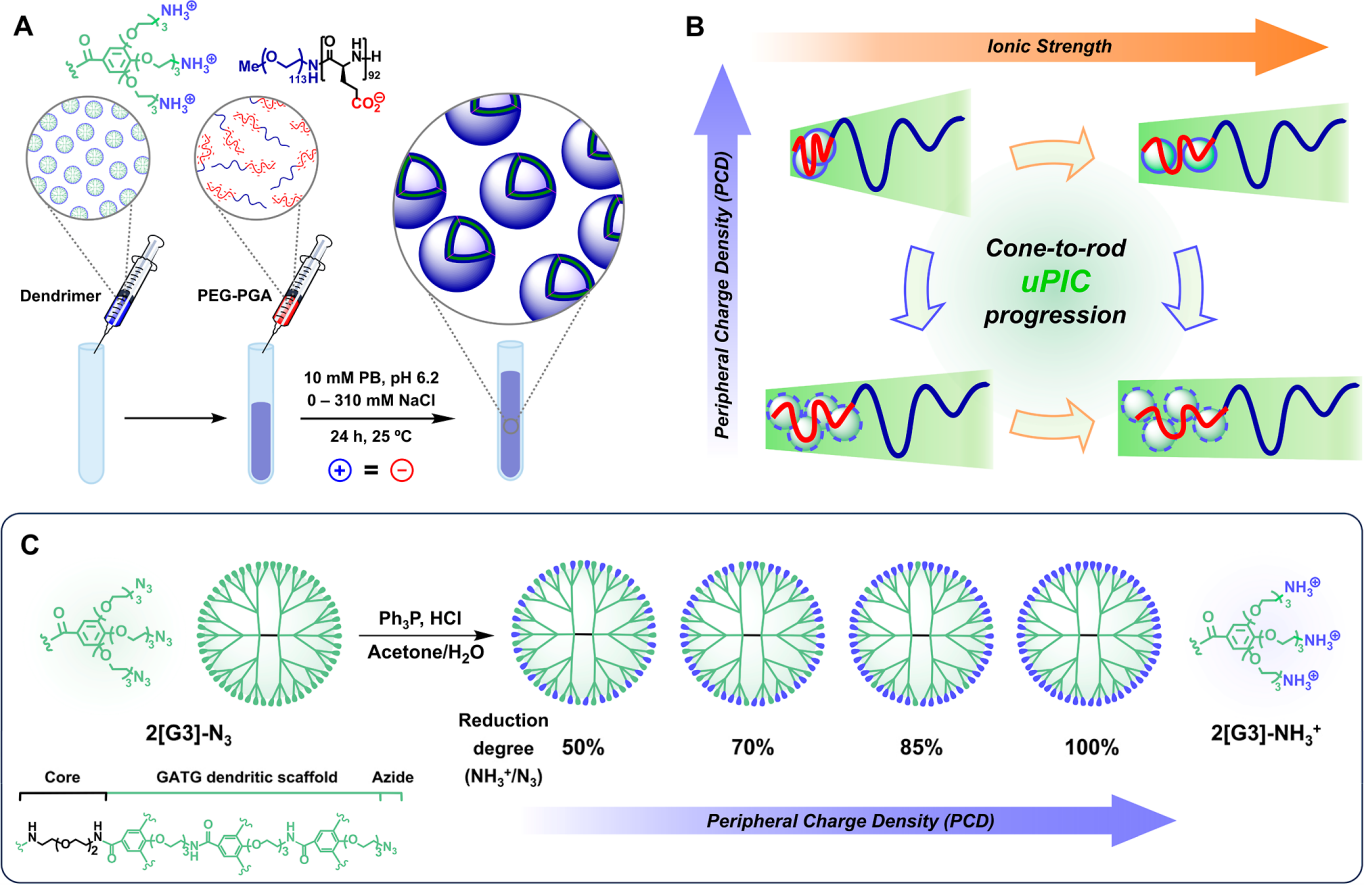
Preparation of dendritic PIC assemblies from a cationic dendrimer and an anionic PEG–PGA copolymer [(poly­(ethylene glycol)-*block*-poly- *l* -glutamic acid] (**A**). The PCD of the dendrimer and the ionic strength of the medium allow tuning the uPIC architecture and the size and morphology of PIC assemblies. Either reducing the PCD (from top down) or increasing the ionic strength (from left to right) favors a cone-to-rod progression of the uPIC leading to larger PICsomes (**B**). Synthesis of dendrimers with variable PCD by partial reduction of the terminal azides of 2­[G3]-N_3_ (**C**).

## Results and Discussion

### On-Demand Size of PICsomes by Tuning the Peripheral Charge Density of a Dendrimer and the Ionic Strength of the Medium

In the quest for innovative strategies to tune the uPIC architecture of dendritic PICsomes with a single G, we turned our attention to the dendrimer PCD and ionic strength of the medium (Figure 2). It was hypothesized that reducing the dendrimer charge density (while maintaining size) would increase the number of dendrimers recruited by an oppositely charged block copolymer in the construction of the uPIC, forcing its rod-like character and the production of larger PICsomes. Also, it was envisaged that increasing the ionic strength of the medium would weaken the electrostatic interaction between the charged dendrimers and block copolymer, leading to loosened, more rod-shaped uPICs, and larger PICsomes. Indeed, it is known that upon increasing ionic strength, PIC assemblies swell before progressively disassembling above a critical ionic strength.^61–63^ Our aim was to take advantage of the high salt-persistency of dendritic PICsomes^43,56^ to fine-tune their size.

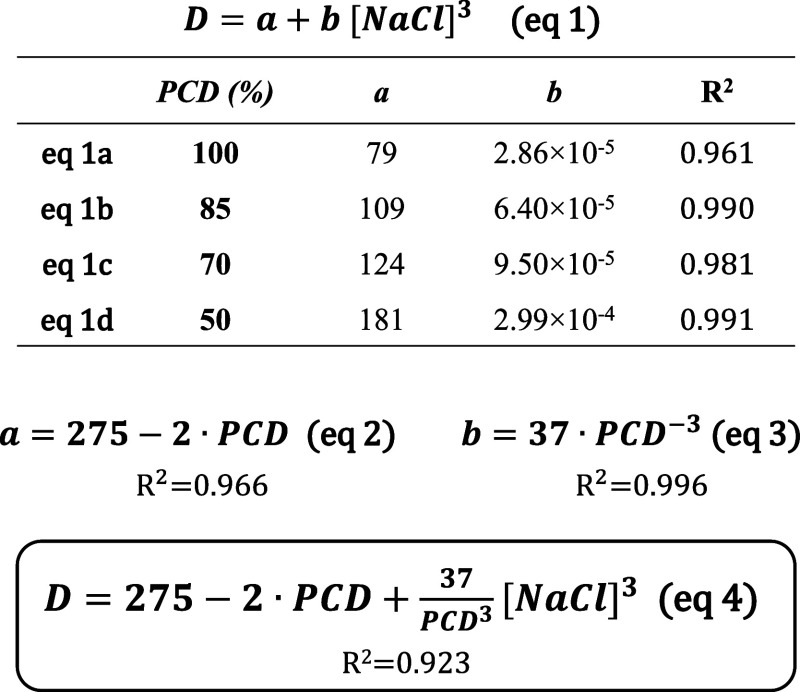



To test this hypothesis, we relied on PICsomes derived from 2­[G3]-N_3_, a small dendrimer with 54 peripheral azide groups (Figure 2C and Schemes S1 and S2).^43^ Reduction of 2­[G3]-N_3_ under Staudinger conditions (Ph_3_P, acetone/H_2_O) afforded 2­[G3]-NH_3_^+^ with a fully cationic surface (54 ammonium groups). Mixing 2­[G3]-NH_3_^+^ with PEG–PGA as oppositely charged block copolymer (PEG_5k_, poly- *l* -glutamic acid block with DP 92) in 10 mM phosphate buffer (PB) pH 6.2, 150 mM NaCl resulted in PICsomes of ca. 200 nm of diameter by dynamic light scattering (DLS) (Figures 2A, 3A,B).^43^ Since azide reduction can be easily controlled by the stoichiometry of Ph_3_P (Figure 2C), partial reduction of 2­[G3]-N_3_ was used to synthesize 2­[G3]-N_3_/NH_3_^+^ dendrimers with variable PCD to evaluate its influence and that of the ionic strength on the size of PICsomes. Four 2­[G3]-N_3_/NH_3_^+^ dendrimers with PCD (reduction degrees) 50, 70, 85, and 100% (equivalent to 27/27, 16/38, 8/46, and 0/54 azide/ammonium groups) were obtained in excellent yields. The reduction process was easily monitored by IR and ^1^H NMR (see the Supporting Information). IR showed a progressive disappearance of the characteristic intense azide band at ca. 2100 cm^–1^. Reduction degrees were determined by integration of the protons in α position to the terminal ammonium groups (3.30–3.10 ppm) relative to the 52 aromatic protons (7.40–7.10 ppm) and 156 methylene protons in alpha to the amide groups (4.40–4.10 ppm). Neither the PCD nor the ionic strength showed any effect on the hydrodynamic size of the dendrimers as determined by DLS (see the Supporting Information). PIC assemblies were prepared with the four dendrimers and PEG–PGA under a stoichiometric charge ratio in 10 mM phosphate buffer pH 6.2, supplemented with increasing concentrations of NaCl (Figure 3, Tables S1 and S2). Assemblies were denoted as PIC_PCD‑[NaCl]_, where PCD refers to the peripheral charge density of the dendrimer (50, 70, 85, and 100%) and [NaCl] to the salt concentration of the medium (0–310 mM). The formation of the assemblies was followed by DLS at 25 °C for 24 h. Size steadily increased with time before reaching a constant value, with larger assemblies requiring longer times to stabilize. Monomodal size distributions without secondary populations were consistently obtained with polydispersity indexes (PDI) below 0.2–0.3, calculated using the cumulants algorithm. Figures 3A,B, S1–S7 and Table S2 shows DLS data recorded after 5 and 24 h.

**Figure 3:**
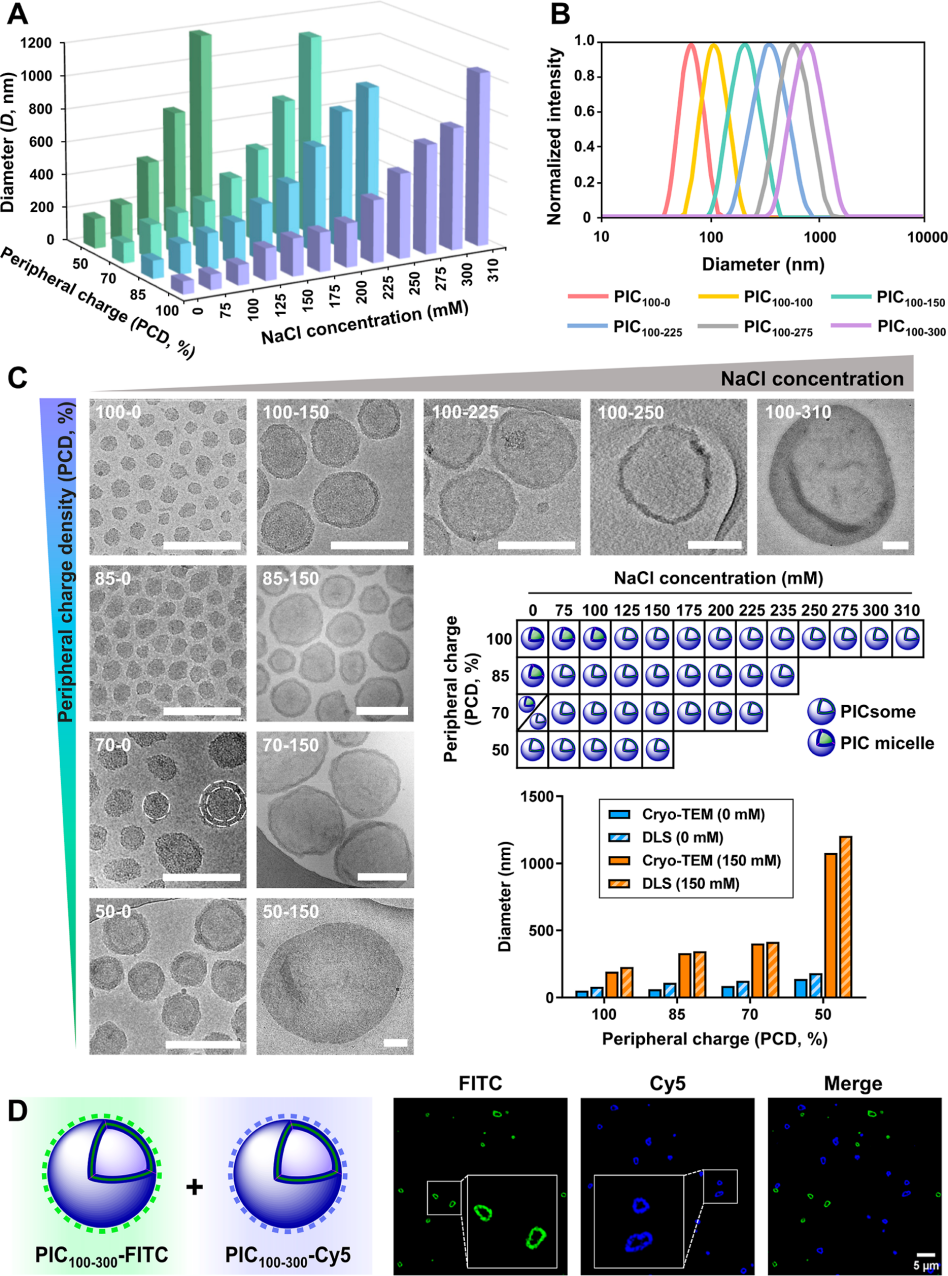
DLS mean hydrodynamic diameter (**A**) and size distribution (**B**) of PIC assemblies prepared at increasing ionic strengths from PEG–PGA and 2­[G3]-N_3_/NH_3_^+^ dendrimers with variable PCD (DLS recorded after 24 h). Cryo-TEM images of representative PIC assemblies (scale bar 200 nm), summary diagram of the PIC morphology (micelle versus vesicle) obtained by cryo-TEM, and comparison of mean diameters by cryo-TEM and DLS (**C**). CLSM images of fluorescently labeled PICsomes PIC_100–300_-FITC (green) and PIC_100–300_-Cy5 (blue) after 24 h of mixing (**D**).

The effect of the PCD of the dendrimer on the size of the assemblies was first analyzed with a series of PICsomes prepared with the four dendrimers in 10 mM PB supplemented with 150 mM NaCl. A size increase from ca. 200 to 1200 nm was observed when reducing the PCD in agreement with the expected increase in the number of dendrimers recruited within the uPIC. Complexation was then assessed for the four dendrimers at increasing NaCl concentrations. An increase in size with the ionic strength of the medium was observed from ca. 80–180 nm (depending on the PCD) to above the micron range, consistent with the involvement of loosened, more rod-shaped uPICs. A critical NaCl concentration was shown for each dendrimer, above which monodispersity was not guaranteed after 24 h. Despite variations in this critical concentration with the dendrimer PCD (from 150 mM NaCl for 50% PCD to 310 mM NaCl for 100% PCD), no significant differences were observed in the size of the largest PICsomes achieved. As seen for 150 mM NaCl, reducing the PCD also resulted in an increase in size for each of the other NaCl concentrations studied.

Complexation was challenged by preparing the assemblies in the absence of salt, followed by dialysis against any NaCl concentration of interest, a strategy envisioned to accelerate the production of PICsomes with on-demand size. No significant differences were obtained between both methods, which highlights the robustness and fidelity of the process (Figure S8). Interestingly, the assemblies could be cross-linked with EDC and dialyzed against 10 mM PB pH 7.4, 150 mM NaCl (physiological conditions) to yield stable PICs that kept their original size. Despite the high charge of the dendrimers and PEG–PGA, *z*-potential values for PIC were close to zero, in agreement with the charge stoichiometry of the constituents and the presence of the PEG palisade (see the Supporting Information).

For the four dendrimers, the plots of the mean hydrodynamic diameters (*D*) of the PIC assemblies versus the NaCl concentration ([NaCl], mM) were fitted to cubic power functions with intercepts equal to the *D* values obtained in the absence of NaCl (eqs 1a–d, Figure S9). Interestingly, the intercepts and coefficients in eqs 1a–d increase when reducing the PCD of the dendrimer, with variations that fit a straight line and the inverse of a cubic power function, respectively (eqs 2 and 3, Figure S10). Substituting eqs 2 and 3 into eq 1 afforded eq 4, which allows predicting the hydrodynamic diameter (*D*) of PIC assemblies with on-demand size by simply adjusting the PCD of the dendrimer and NaCl concentration of the medium (coefficient of determination, *R*-squared, *R* ^2^ = 0.923). A detailed description of the derivation of eq 4 can be found in the Supporting Information. Comparison of experimental hydrodynamic diameters and calculated values using eq 4 shows a high level of accuracy (Figure S11). A diameter calculator based on eq 4 is provided in the Supporting Information as an Excel spreadsheet to allow the estimation of *D*, given the PCD of the dendrimer and the NaCl concentration. Overall, the PCD of the dendrimer and the ionic strength of the medium reveal as privileged tools for the efficient size tuning of PIC assemblies via a precise dendrimer-to-PIC hierarchical transfer of structural information. Such an organized structure allows the size variation to be mathematically modeled by eq 1–4. We envisage that similar strategies can be applied generally to model nanoscale properties in alternative systems,^44^ where information is hierarchically transferred bottom-up from the (macro)­molecular building blocks to the structure and properties of the final nanostructures. PICsomes larger than one micron were readily obtained for evaluation as synthetic cells, while reducing the overall synthetic effort of the dendrimer endeavor to preparing a single G.

### Characterization of PICsomes by Cryo-TEM and Confocal Microscopy

A detailed structural analysis of the assemblies performed by cryo-transmission electron microscopy (cryo-TEM) revealed spherical particles with size distributions only slightly smaller than those obtained by DLS (Figures 3C and S12). Interestingly, while small PIC form ordered superstructures in the thin ice layer as described by Velders,^64^ larger assemblies embedded in a thicker ice are randomly distributed throughout the grid hole. A morphological change from compact micellar structures to larger vesicles (with uniform lamella of thickness of 22 ± 2 nm) was observed when increasing the ionic strength of the medium or reducing the PCD of the dendrimer, in line with the pursued cone-to-rod uPIC progression (Figure 3C). This micelle-to-vesicle transition, which occurs around 150 nm, is particularly evident for PIC_70–0_ that shows both morphological structures by cryo-TEM: a major population of micelles with an average size of 68 ± 11 nm accompanied by a minor proportion of larger PICsomes of 111 ± 12 nm (Figure S12). The presence of micelles at low ionic strengths causes deviations in the fittings of eqs 1a-c (Figure S9) which explain the overestimated hydrodynamic diameters predicted for the larger assemblies (Figure S11).

Recognizing the potential utility of micron-sized PICsomes as synthetic cells, an analysis by confocal laser scanning microscopy (CLSM) was done. The vesicular structure of PIC_100–300_ was unambiguously determined using a fluorescently labeled version of the PICsome prepared from PEG–PGA-FITC, a block copolymer incorporating fluorescein isothiocyanate at the terminal amino group of the PGA block. Figure 3D shows a well-defined vesicular organization with a green membrane (FITC), indicative of a lamellar assembly of the polymers. The integrity of the PICsomes over time and the ability of different PICsome populations to coexist was assessed using two fluorescently labeled PICsomes (FITC and Cyanine 5, the latter prepared with PEG–PGA-Cy5). After 24 h of mixing, no fusion or migration of macromolecular components between PICsomes was observed by confocal microscopy (Figure 3D), a hallmark of effective compartmentalization. The permeability of the PIC membrane to small molecules, and hence the ability of PICsomes to chemically communicate, was then assessed via an enzymatic cascade reaction.

### Embedding Enzymes in the Membrane of Dendritic PICsomes

The enzymatic cascade composed of glucose oxidase (GOX, 160 kDa, pI 4.2; pI is the isoelectric point) and horseradish peroxidase (HRP, 44 kDa, pI 9.0) was selected to demonstrate the feasibility of PICsomes to emulate life-like technologies such as enzyme encapsulation and chemical communication. Both enzymes were fluorescently labeled with Alexa Fluor 488 (GOX-AF488) and Cy5 (HRP-Cy5) and separately encapsulated in complementary fluorescent PICsomes: GOX-AF488@PIC_100–300_-Cy5 and HRP-Cy5@PIC_100–300_-FITC. To this end, the enzymes were premixed with the equally charged PIC component (cationic HRP with dendrimer/anionic GOX with PEG–PGA) before PICsome formation (DLS histograms of the PICsomes in Figure S13). The encapsulation efficiency was very high regardless of the molecular weight and pI of the protein, with values of 85 ± 4% for GOX and 62 ± 8% for HRP that exceed those typically found for PICsomes (less than 4%) (Figure S13).^33,35,39,40^ These encapsulation efficiencies account for enzyme loadings (defined as the mass fraction of loaded enzyme relative to enzyme-loaded PICsome) of 28% for GOX and 7.2% for HRP. Remarkably, CLSM experiments showed clean dye colocalization in both PICsomes, indicating a selective enzyme embedding in the membrane (Figure 4), in contrasts to the widely accepted encapsulation of proteins in the vesicle lumen of classical PICsomes.^33–36^ While the interactions that favor protein incorporation into the dendritic PIC layer require further investigation, it is hypothesized that dendrimer rigidity might play a role similar to that of cholesterol and other lipids in regulating a diverse range of protein functions in the cell membrane.^65–67^ Selective protein embedding in the membrane explains the high encapsulation efficiencies observed compared to classical PICsomes, where low encapsulation efficiencies result in the absence of driving forces to sequester proteins in the vesicle aqueous lumen (statistical process).

**Figure 4:**
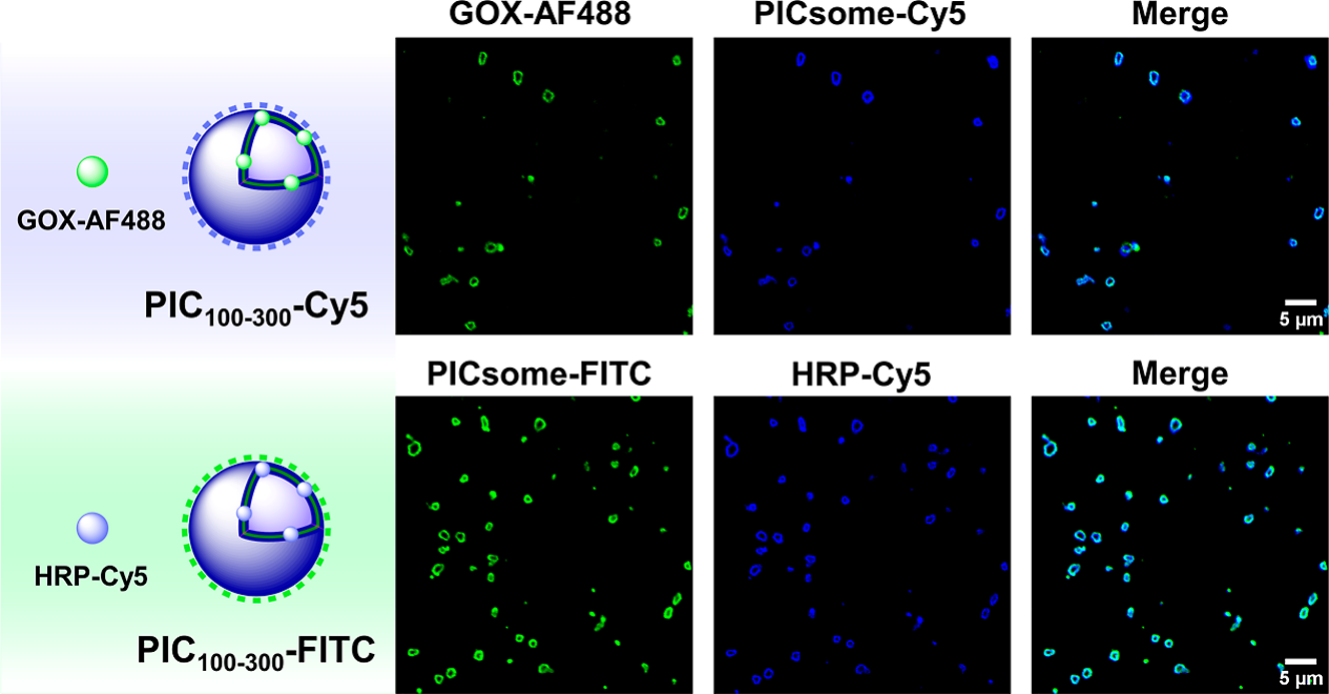
CLSM images of double fluorescently labeled enzyme-loaded PICsomes [GOX-AF488 (green) at PIC_100–300_-Cy5 (blue) and HRP-Cy5 (blue) at PIC_100–300_-FITC (green)] show selective protein localization at the PICsome membrane.

### Chemical Communication between Enzyme-Loaded PICsomes

The potential of enzyme-loaded PICsomes as synthetic cells was assessed with the GOX-HRP enzymatic cascade. In the presence of O_2_, GOX catalyzes the oxidation of β-*D*-glucose to *D*-glucono-1,5-lactone and H_2_O_2_. The latter is used by HRP to oxidize a nonfluorescent/noncolored molecule to a fluorescent/colored one, the detection of which is used to monitor the progress of the enzymatic cascade by confocal microscopy or visible spectroscopy (Figure 5A). Incorporation of the enzymes in different PICsome populations was envisaged to assess their membrane permeability for small substrates (glucose and nonfluorescent/noncolored molecules) and chemical communication (H_2_O_2_). Amplex Red is a nonfluorescent molecule which is oxidized by HRP to give fluorescent resorufin (λ_ex_ 572 nm, λ_em_ 583 nm). The efficiency of the enzymatic cascade was evaluated by CLSM following the appearance of the fluorescent signal of resorufin (red) after addition of glucose to a mixture of Amplex Red, GOX-AF488@PIC_100–300_ (green), and HRP-Cy5@PIC_100–300_ (blue) (Figure 5A,B). The visualization of the resorufin fluorescence in the membrane of both PICsome populations after only 5 min of reaction time confirmed a fast production of H_2_O_2_, chemical communication between PICsomes, and oxidation of Amplex Red. Also, an efficient membrane semipermeability for both substrates and products. Although resorufin is selectively produced at the HRP-loaded PICsome, it can easily cross the membrane and equilibrate throughout the entire PICsome population as described by van Hest with coacervate synthetic cells.^68^ No enzyme equilibration between PICsome populations was seen. Of note, control experiments carried out under identical conditions in the absence of either GOX- or HRP-loaded PICsomes did not produce resorufin fluorescence, confirming the necessity of both populations for a successful enzymatic cascade (Figure S14).

**Figure 5:**
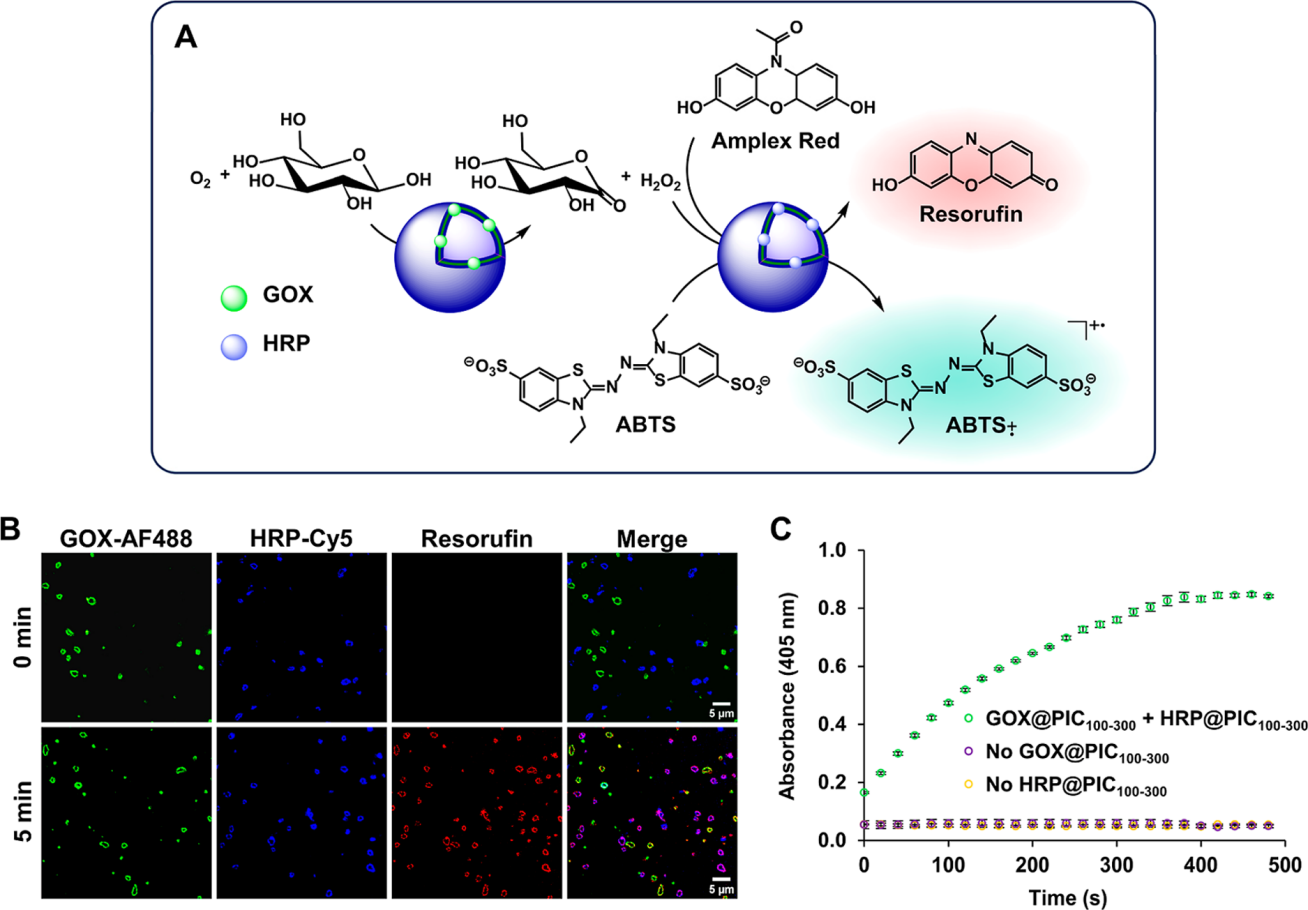
Scheme of the GOX-HRP enzymatic cascade with two independent PICsome populations (**A**). CLSM images of the reaction between GOX-AF488@PIC_100–300_ (green) and HRP-Cy5@PIC_100–300_ (blue) in the presence of Amplex Red before (0 min) and after (5 min) addition of glucose. The production of resorufin (red) confirms the efficient chemical communication between PICsomes (**B**). The progress of the enzymatic cascade was studied using ATBS as HRP substrate by monitoring the absorbance of the ABTS radical cation (405 nm) (**C**).

It is known that Amplex Red is photoxidized to resorufin upon exposure to light in a process initiated by trace amounts of resorufin present in Amplex Red. Although this side reaction can be managed when using single fluorescence measurements as in CLSM, Amplex Red is not recommended for experiments where continuous measurements are needed.^69^ For this reason, ABTS (2,2′-azino-bis­(3-ethylbenzothiazoline-6-sulfonic acid)) was chosen as HRP substrate to analyze the time-dependence of the enzymatic cascade (Figure 5A). The one-electron oxidation of ABTS by H_2_O_2_ in the presence of HRP produces the ABTS radical cation, a colored product that absorbs at 405 nm. Continuous measurement of the increase in absorbance due to the ABTS radical cation allowed monitoring of the reaction progress of a mixture of GOX@PIC_100–300_ and HRP@PIC_100–300_ in the presence of glucose and ABTS (0.93 μM GOX and 0.79 μM HRP). Less than 7 min after the addition of glucose to initiate the reaction, the absorbance reached a plateau, indicating the completion of the process (Figure 5C). The cascade proceeded with a remarkable 85 ± 3% activity relative to the free enzymes, confirming that embedded enzymes retain high catalytic activity. As above, when the reaction was performed in the absence of any of the enzyme-loaded PICsomes, no absorbance increase was observed.

The stability conferred by the dendritic PICsome membrane on embedded proteins was then assessed. First, a comparison by DLS showed no change in the histogram and correlation function of a mixture of GOX- and HRP-loaded PICsomes after 24 h of incubation at 37 °C with proteinase K (p*K*, up to 200 μM), demonstrating a notable resistance of the PICsome membrane to proteolytic degradation (Figure S15). Then, the enzymatic activity of PICsomes incubated with 10 μM p*K* was assessed using ABTS (0.93 μM GOX and 0.79 μM HRP). Gratifyingly, GOX@PIC_100–300_ and HRP@PIC_100–300_ showed an excellent level of protection against proteolysis after 24 h, retaining 86 ± 2% of the enzymatic activity of the control. In contrast, a solution of free GOX and HRP incubated with 10 μM p*K* under identical conditions showed no enzymatic activity (see the Supporting Information). Similarly, a 70% loss of enzymatic activity after only 5 min of treatment with trypsin has been reported for nondendritic PICsomes, where enzymes are not embedded but accumulated on the PIC membrane by electrostatic and/or hydrophobic interactions.^40^ These results demonstrate that proteases hardly access the membrane of dendritic PICsomes, which protects embedded proteins from proteolytic degradation and ensures their long-term stability and activity. Finally, since replicating cellular functions in biological environments requires synthetic cells to be stable and maintain their structure under physiological conditions,^7^ the stability of PICsomes was assessed in cell culture. No variation in the size and size distribution was observed by DLS after 24 h (Figure S16). Overall, our results pave the way for the wider use of PICsomes as synthetic cells with promising applications in advanced therapies,^70^ tissue engineering and regenerative medicine,^71–74^ and compartmentalized enzymatic bioreactors.^75^ ^,^

## Conclusions

Despite high permeability for small molecules, the small size (typically sub-200 nm) and low encapsulation efficiency of enzymes (less than 4%) have limited the development of polyion complex vesicles (PICsomes) as synthetic cells. Recognizing that the size of PICsomes depends on the architecture and self-assembly of the monomeric unit PIC (uPIC, the minimum neutral assembly formed from oppositely charged species in the early stages of the PIC growth), we describe that either reducing the peripheral charge density (PCD) of a dendrimer (more dendrimers recruited within the uPIC) or increasing the ionic strength of the medium (weaker electrostatic interactions) enforces the rod-like character of the uPIC and the production of larger PICsomes, with on-demand size beyond the micron range. Interestingly, fitting the variation of the hydrodynamic diameters to the PCD and salt concentration allowed derivation of eqs 1–4, which provide size estimates with a high degree of accuracy. The ability of these PICsomes to emulate life-like technologies was assessed with an enzymatic cascade. Selective enzyme embedding in the membrane was shown by confocal microscopy with efficiencies up to 85%, as opposed to the low protein encapsulation in the lumen of classical PICsomes. While the interactions that favor protein incorporation into the dendritic PIC layer require further investigation, it is hypothesized that the dendrimer rigidity might play a role similar to that of cholesterol and other lipids in the cell membrane. Fast enzymatic cascade and communication between PICsome populations were demonstrated, with embedded enzymes retaining high catalytic activity (85 ± 3% relative to the free enzymes), even in the presence of proteases. Overall, by controlling the PCD of a single dendrimer generation (G) and the ionic strength of the medium, PICsomes beyond the micron range were readily obtained through a dendrimer-to-uPIC hierarchical transfer of structural information. These PICsomes exhibited high encapsulation efficiency, enzyme protection, and fast chemical communication, marking a significant advance in PICsome technology while paving their way for a wider application in advanced therapies, tissue engineering, regenerative medicine, and compartmentalized enzymatic bioreactors.

## Experimental Section

### Materials

Ph_3_P was purchased from Sigma-Aldrich and recrystallized from ethanol. *N*-acetyl-3,7-dihydroxyphenoxazine (Amplex Red) was supplied by Biosynth. 2,2′-azino-bis­(3-ethylbenzothiazoline-6-sulfonic acid) (ABTS) was purchased from Alfa Aesar. Peroxidase from Horseradish type VI (HRP) and Glucose Oxidase (GOX) from were supplied by Sigma-Aldrich. Proteinase K was purchased from Glentham Life Sciences. All other chemicals were purchased from Sigma-Aldrich or Thermo Fisher Scientific. All solvents were HPLC grade, purchased from Scharlab, Sigma-Aldrich, or Acros Organics. DMSO was dried under 4 Å molecular sieves H_2_O of Milli-Q grade was obtained from a Millipore water purification system. 2­[G3]-N_3_ was synthesized as previously reported.^43^ Methoxypoly­(ethylene glycol)-*block*-poly­(l-glutamic acid sodium salt) PEG–PGA (*M*_*n*_ 19000; *M*_*n*_ of PEG 5004, DP of PGA 92 by NMR) was purchased from Alamanda Polymers. Cy5-NHS and AF488-NHS were purchased from Lumiprobe GmbH.

### Instrumentation

#### NMR Spectroscopy

NMR spectra were recorded on Bruker DRX 500 MHz spectrometers. Chemical shifts (δ) are reported in ppm relative to the residual solvent peak (3.31 ppm for CD_3_OD). MestReNova 14.2.2 software (Mestrelab Research) was used for spectral processing.

#### Infrared Spectroscopy

FT-IR spectra were recorded on a Bruker Vertex 70v, using an ICs pellet transmission method with 32 scans and a resolution of 4 cm^–1^. Spectra were processed using OPUS 7.8 software.

#### UV–Vis Spectroscopy

UV–vis spectra were recorded on a Jasco V-750 spectrometer.

#### Determination of pH Values

pH values were measured with a portable pH-meter (Crison PH25) connected to a glass electrode (Crison 52 09).

#### Dialysis and Ultrafiltration

Dialysis was performed with 18 mm Spectra/Por 6 MWCO 1 kDa and 10 mm Spectra/Por Biotech Cellulose Ester MWCO 1000 kDa membranes from Spectrum Labs. Ultrafiltrations were performed on Millipore Amicon stirred cells with Amicon YM3 (MWCO 3 kDa) and YM5 (MWCO 5 kDa) regenerated cellulose membranes under a 5 psi N_2_ pressure.

#### Confocal Laser Scanning Microscopy

Confocal images were captured on an Andor Dragonfly spinning disk confocal system mounted on a Nikon TiE microscope equipped with a Zyla 4.2 PLUS sCMOS digital camera (Andor, Oxford Instruments). Samples were excited with three different lasers (488, 561, and 637 nm) and the emitted fluorescence was collected by the filter wheel (525/50 nm, 620/50 nm, and 725/40 nm) with appropriate combinations of them. Images were taken with 100× magnification objective. All the images were processed with ImageJ software (version 1.51j8).

## Supplementary Material





## Notes

Experimental procedures, characterization, and derivation of eq 4 (PDF) (PDF)
Diameter Calculator based on eq 4 (Excel) (XLSX)
