# Supplementary material for: From Nano to Micro Polyion Complex Vesicles: Synthetic Cells with Membrane-Embedded Enzymes
Source: ACS Appl Mater Interfaces. 2025 Aug 11;17(33):47426–35. doi: 10.1021/acsami.5c11988 (PMC12371685; doi:10.1021/acsami.5c11988)
Supplement: Supplementary file 1 [file am5c11988_si_001.pdf]

Supporting Information

From Nano to Micro Polyion Complex Vesicles: Synthetic Cells with Membrane-Embedded Enzymes

*Celia Jimenez-Lopez, Roi Lopez-Blanco, Iria Esperon-Abril, and Eduardo Fernandez-Megia**

Centro Singular de Investigación en Química Biolóxica e Materiais Moleculares (CIQUS), Departamento de Química Orgánica, Universidade de Santiago de Compostela, Jenaro de la Fuente s/n, 15782 Santiago de Compostela, Spain.

*Email: ef.megia@usc.es

Table of Contents

1. Synthesis and Characterization S3

2. Preparation of PIC Assemblies S14

3. Dynamic Light Scattering: Size and Stability of PIC S15

4. Fitting the Size Variation of PIC with the NaCl Concentration and the Peripheral Charge Density (PCD) of the Dendrimer: Derivation of Eq 4 S25

5. Measurement of Z-Potential S28

6. Cryo-Transmission Electron Microscopy (cryo-TEM) S29

7. Encapsulation of Enzymes S31

8. Enzymatic Cascade Assays S33

9. Stability in Cell Culture Medium S35

10. References S36

1. Synthesis and Characterization

Working with Azides – WARNING!

For warnings on working with azides, readers are referred to ref 1. For organic azides to be manipulable or non-explosive, the "Smith's rules" must be followed: i) the number of nitrogen atoms (N_N) must not exceed that of carbon (N_C), and ii) $(N_C + N_O)/N_N \geq 3$. Similarly, the "rule of six" indicates that six carbons (or other atoms of about the same size) per azide provides sufficient dilution to render an organic azide relatively safe.[2,3] All organic azides in this report follow these rules and have proven to be stable in our hands.

Reduction of dendrimers (General Procedure I)

Ph_3P was added to a solution of 2[G3]-N_3 in acetone/H_2O (10:1, 0.1 M per azide) and the mixture was stirred at rt for 24 h. Then, 3 M HCl (2 equiv per amino group) was added and the solvent was evaporated. The crude product was dissolved in H_2O (5 mL) and the solution was filtered through a cotton plug and washed with $CHCl_3$ (5 × 30 mL). The aqueous phase was lyophilized to afford 2[G3]-N_3/NH_3^+ dendrimers with different reduction degrees as pale-yellow foams. Reduction degrees were determined by ^{1}H NMR integration of the protons in alpha position to the terminal ammonium groups (3.30-3.10 ppm) relative to the 52 aromatic protons (7.40-7.10 ppm) and 156 methylene protons in alpha to the amide groups (4.40-4.10 ppm).

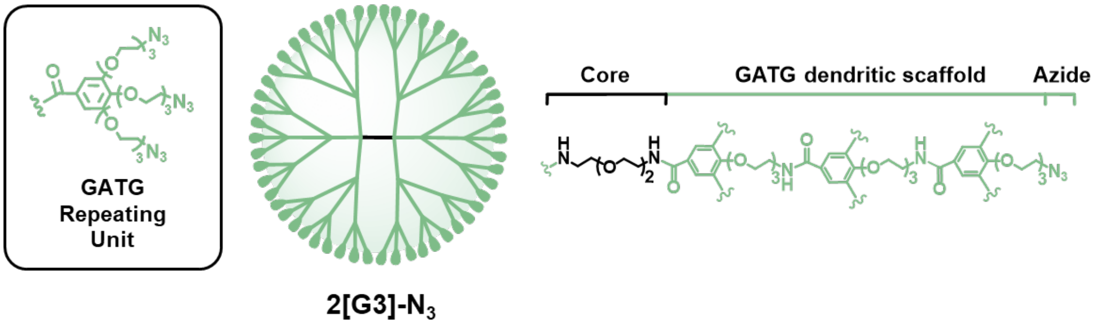

Scheme S1. Structure of 2[G3]-N_3.

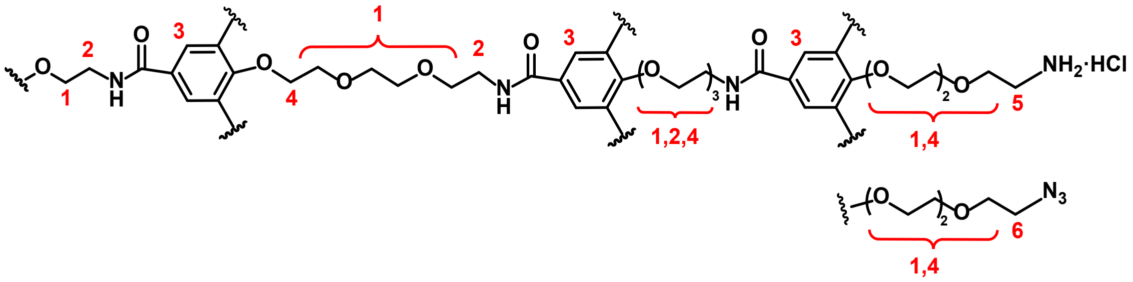

Scheme S2. Structure of 2[G3]-N_3/NH_3^+ dendrimers.

2[G3]-(NH_2·HCl)$_{54}$. 2[G3]-(NH_2·HCl)$_{54}$ (38.4 mg, 97%) was obtained from 2[G3]-N_3 (40.0 mg, 2.5 μmol) and Ph_3P (43.2 mg, 0.16 mmol) following General Procedure I.

^{1}H NMR (500 MHz, CD_3OD) δ: 7.40-7.10 (m, 52H), 4.40-4.10 (m, 156H), 4.01-3.55 (m, 684H), 3.30-3.10 (m, 108H). IR (ICs): 2874, 1639, 1111 cm^{-1}.

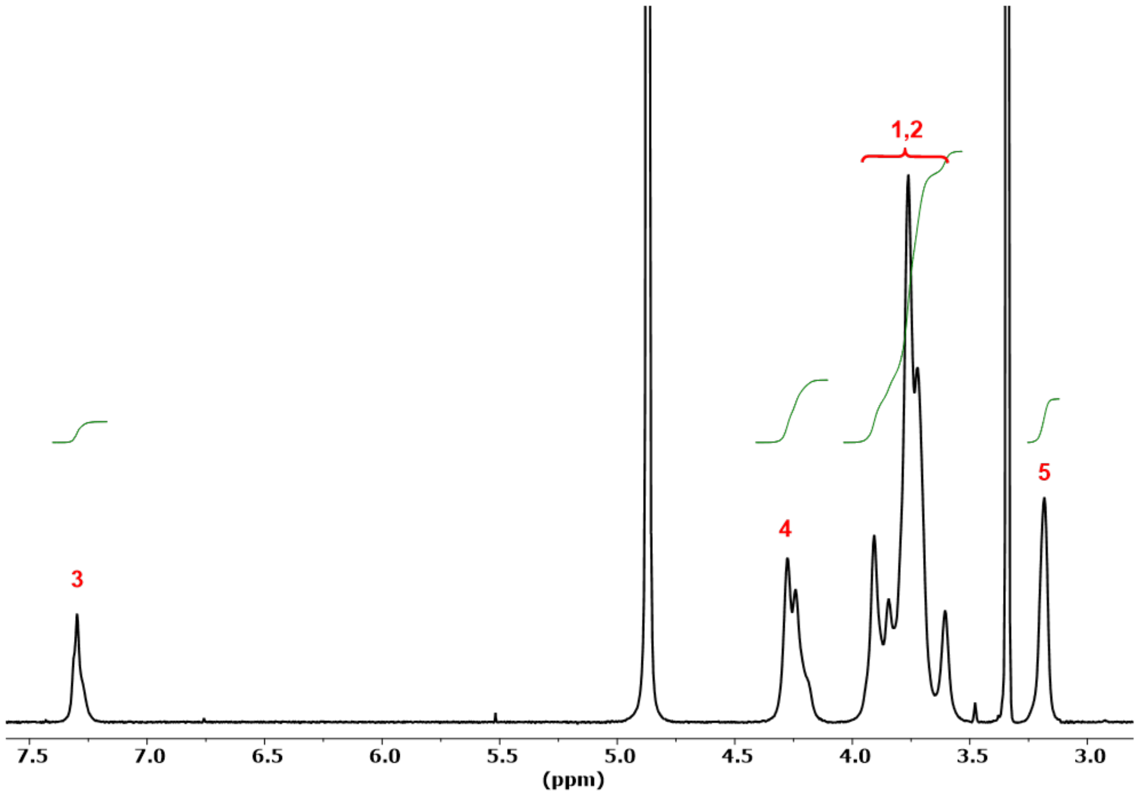

^{1}H NMR spectrum (CD_3OD, 500 MHz) of 2[G3]-(NH_2·HCl)$_{54}$.

2[G3]-$(N_3)_8(NH_2{\cdot}HCl)_{46}$. 2[G3]-$(N_3)_8(NH_2{\cdot}HCl)_{46}$ (41.7 mg, 92%) was obtained from 2[G3]-N_3 (44.1 mg, 2.8 μmol) and Ph_3P (33.8 mg, 0.13 mmol) following General Procedure I.

^1H NMR (500 MHz, CD_3OD) δ: 7.40-7.10 (m, 52H), 4.40-4.10 (m, 156H), 4.01-3.55 (m, 684H), 3.30-3.10 (m, 92H). IR (ICs): 2874, 2106, 1638, 1115 cm^{-1}.

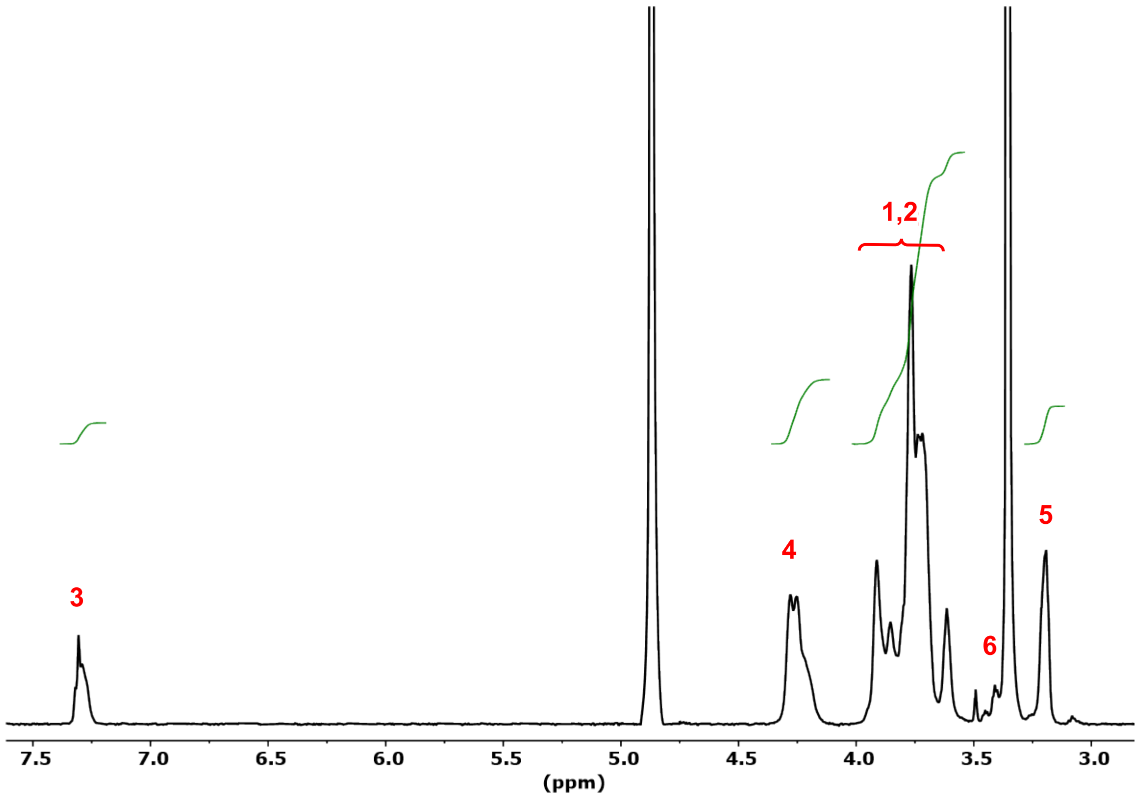

^1H NMR spectrum (CD_3OD, 500 MHz) of 2[G3]-$(N_3)_8(NH_2{\cdot}HCl)_{46}$.

2[G3]-$(N_3)_{16}(NH_2 \cdot HCl)_{38}$. 2[G3]-$(N_3)_{16}(NH_2 \cdot HCl)_{38}$ (27.6 mg, 94%) was obtained from 2[G3]-N_3 (29.8 mg, 1.9 µmol) and Ph_3P (18.9 mg, 72.0 µmol) following General Procedure I.

^1H NMR (500 MHz, CD_3OD) δ: 7.40-7.10 (m, 52H), 4.40-4.10 (m, 156H), 4.01-3.55 (m, 684H), 3.30-3.10 (m, 76H). IR (ICs): 2873, 2104, 1639, 1115 cm^{-1}.

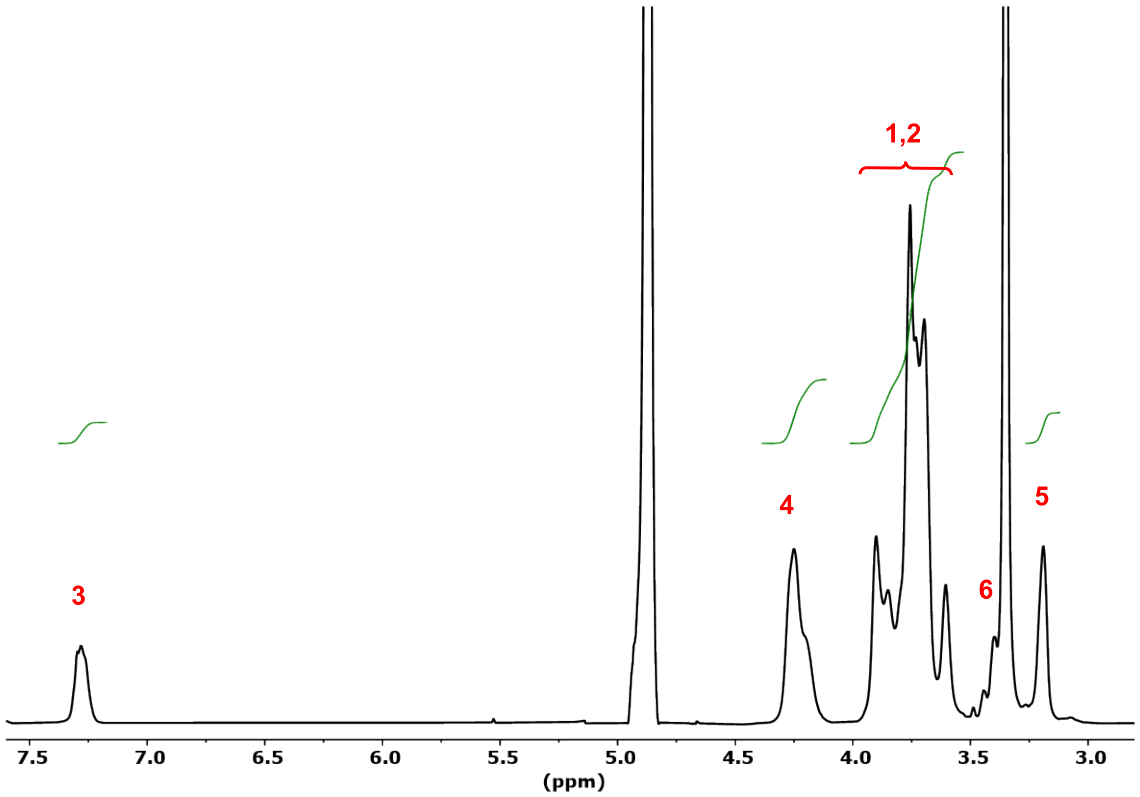

^1H NMR spectrum (CD_3OD, 500 MHz) of 2[G3]-$(N_3)_{16}(NH_2 \cdot HCl)_{38}$.

2[G3]-(N_3)$_{27}$($NH_2 \cdot HCl$)$_{27}$. 2[G3]-(N_3)$_{27}$($NH_2 \cdot HCl$)$_{27}$ (50.9 mg, 93%) was obtained from 2[G3]-N_3 (52.1 mg, 3.3 μmol) and Ph_3P (23.4 mmol, 89.4 μmol) following General Procedure I.

^{1}H NMR (500 MHz, CD_3OD) δ: 7.40-7.10 (m, 52H), 4.40-4.10 (m, 156H), 4.01-3.55 (m, 684H), 3.30-3.10 (m, 54H). IR (ICs): 2872, 2104, 1645, 1115 cm^{-1}.

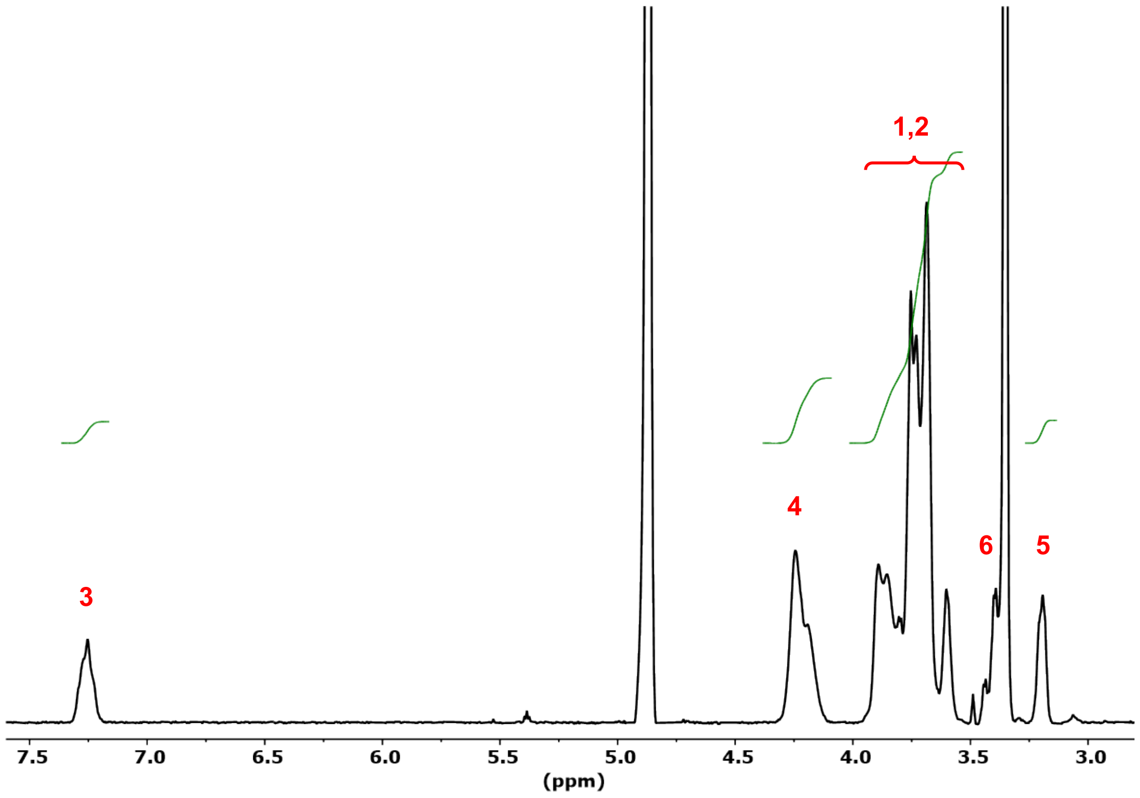

^{1}H NMR spectrum (CD_3OD, 500 MHz) of 2[G3]-(N_3)$_{27}$($NH_2 \cdot HCl$)$_{27}$.

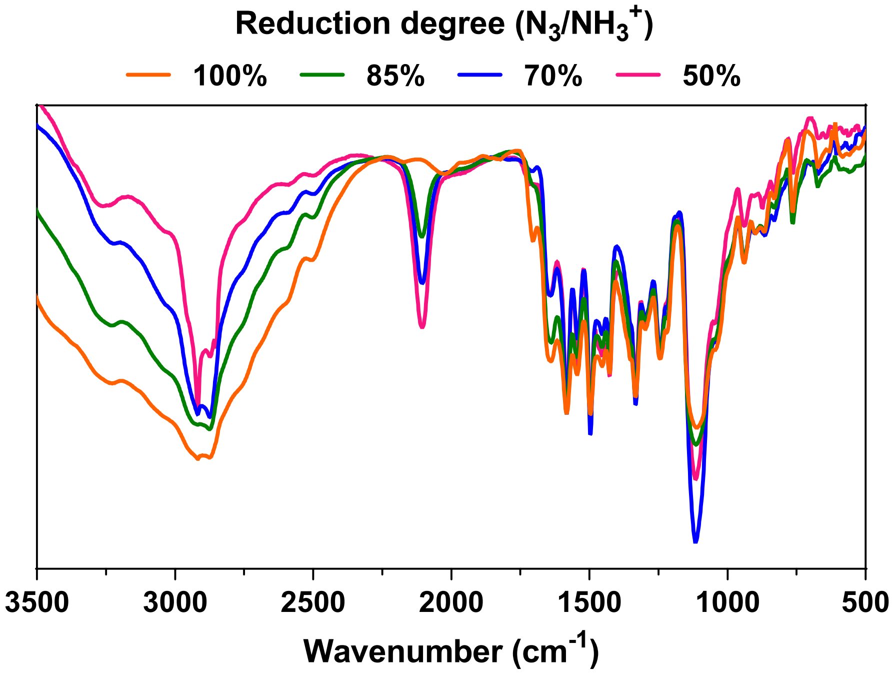

IR (ICs) spectra of 2[G3]-N_3/NH_3^+ dendrimers with reduction degrees 50, 70, 85, and 100%.

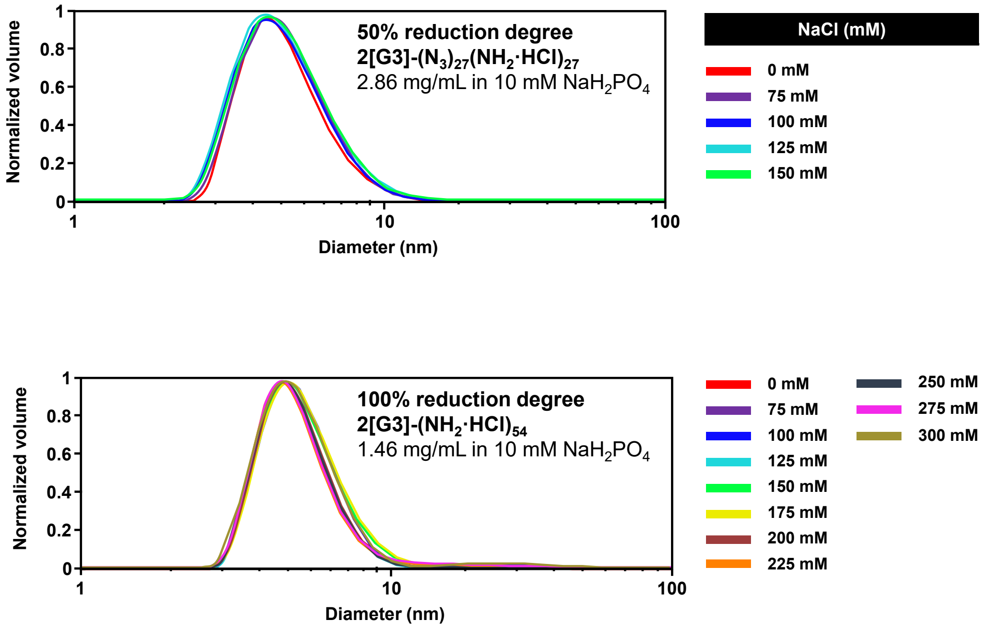

DLS size distribution of 2[G3]-N_3/NH_3^+ dendrimers in 10 mM NaH_2PO_4 supplemented with increasing concentrations of NaCl.

Fluorescent labelling of PEG-PGA

PEG-PGA-FITC. A solution of fluorescein 5(6)-isothiocyanate (8.2 mg, 21 μmol) in dry DMSO (0.4 mL) was added to a solution of PEG-PGA (20.0 mg, 1.1 μmol) in 0.1 M $NaHCO_3$ pH 9 (10.2 mL). After 18 h of stirring at rt protected from light, the reaction mixture was purified by dialysis [1 × 2 L MeOH:H_2O (1:1), 5 × 2.5 L 10 mM Na_2HPO_4, 2 × 1 L H_2O; MWCO 1 kDa, Spectra/Por 6]. After freeze-drying, PEG-PGA-FITC (16.0 mg, 80%) was obtained as a yellow solid. A degree of functionalization of 2% in FITC was determined by absorbance at 490 nm (ε_{490} 73000 $cm^{-1}M^{-1}$ as provided by supplier).

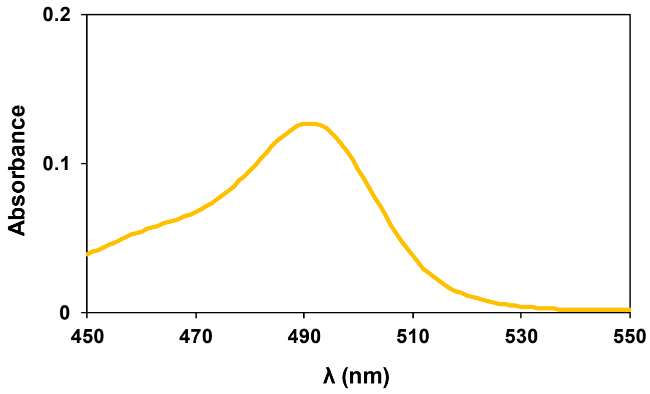

UV-Vis spectrum of PEG-PGA-FITC in H_2O.

PEG-PGA-Cy5. PEG-PGA (25.0 mg, 1.31 μmol) was dissolved in H_2O and 1 M HCl was added till pH 3. The solution was ultrafiltered (YM3) washing with H_2O (4 × 15 mL), aq Et_3N (2.5% v/v, 4 × 15 mL), and H_2O (5 × 15 mL). After freeze-drying, the product was dissolved in dry DMSO (0.65 mL) and added to a solution of Cy5-NHS (2.0 mg, 2.97 μmol) and Et_3N (0.7 μL, 4.90 μmol) in dry DMSO (0.1 mL). After 18 h of stirring at rt protected from light, the reaction mixture was purified by dialysis [2 × 2 L 10 mM Na_2HPO_4, 4 × 2 L 150 mM NaCl, 4 × 2 L H_2O; MWCO 1 kDa, Spectra/Por 6]. After freeze-drying, PEG-PGA-Cy5 (21.0 mg, 84%) was obtained as a blue solid. A degree of functionalization of 62% in Cy5 was determined by absorbance at 644 nm (ε_{644} 250000 $cm^{-1}M^{-1}$ as provided by supplier).

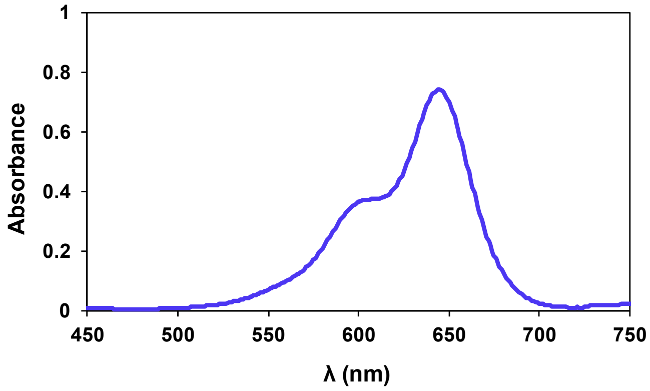

UV-Vis spectrum of PEG-PGA-Cy5 in H_2O.

Fluorescent labelling of enzymes (General Procedure II)

Enzymes were dissolved at 5 mg/mL in 0.1 M $NaHCO_3$ pH 9. A fresh solution of fluorescent dye in dry DMSO was added and the reaction mixture was stirred overnight at rt protected from light. After freeze-drying, the crude products were dissolved in 150 µL of 300 mM NaCl and purified in a PD-10 column (Sephadex G-25 resin) to remove the free dye. Fractions of pure fluorescently labelled enzymes were desalted by dialysis (5 × 500 mL H_2O; MWCO 1 kDa, Spectra/Por 6) and lyophilized. The dye functionalization degrees were determined by measuring the relative UV absorbances of the enzymes and fluorescent dyes using the following extinction coefficients: GOX ε_{280} 267200 $cm^{-1}M^{-1}$, HRP ε_{405} 102000 $cm^{-1}M^{-1}$, AF488 ε_{495} 71800 $cm^{-1}M^{-1}$, Cy5 ε_{646} 250000 $cm^{-1}M^{-1}$.

GOX-AF488. From a solution of AF488-NHS (0.4 mg, 0.51 µmol) in dry DMSO (80 µL) and a solution of GOX (9.0 mg, 0.06 µmol) in 0.1 M $NaHCO_3$ (2.0 mL), GOX-AF488 (8.3 mg) labelled with 2.3 molecules of AF488 was obtained following General Procedure II.

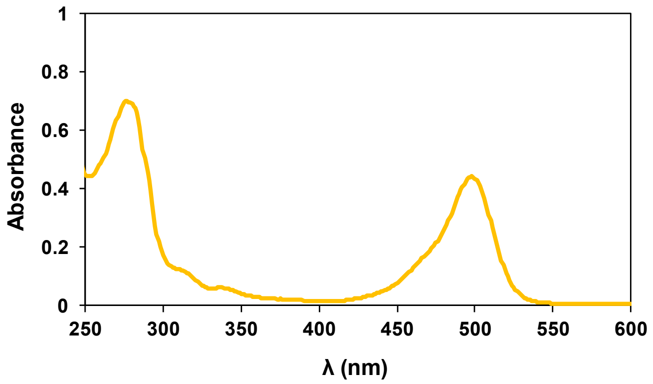

UV-Vis spectrum of GOX-AF488 in H_2O.

HRP-Cy5. From a solution of Cy5-NHS (3.4 mg, 5.1 µmol) in dry DMSO (70 µL) and a solution of HRP (9.0 mg, 0.21 µmol) in 0.1 M $NaHCO_3$ (2.0 mL), HRP-Cy5 (8.1 mg) labelled with 2.2 molecules of Cy5 was obtained following General Procedure II.

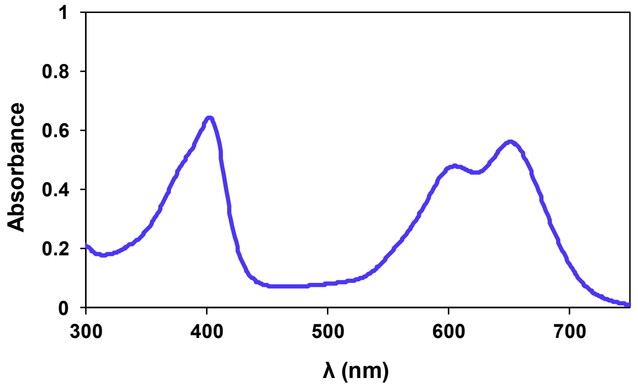

UV-Vis spectrum of HRP-Cy5 in H_2O.

2. Preparation of PIC Assemblies

General notes on PIC assemblies. Solutions of PEG-PGA and dendrimers were aged for 24 h at rt and filtered through 0.45 μm nylon filters immediately before PIC formation. Filtering of PIC before dynamic light scattering (DLS) measurements was avoided to prevent the removal of large aggregates that could obscure the analysis of PIC formation and stability.

Preparation of PIC assemblies (General Procedure III)

PEG-PGA (0.5 mg/mL) was dissolved in 10 mM Na_2HPO_4 supplemented with NaCl at concentrations ranging between 0 and 310 mM. Dendrimers were dissolved in 10 mM NaH_2PO_4/0.1 M HCl (10% v/v) supplemented with NaCl at the same concentrations as above (Table S1). PIC assemblies were prepared by mixing these solutions in a 1:2 ratio, keeping a stoichiometric charge ratio calculated considering full ionization of both polyelectrolytes. The final pH of the solutions was 6.1-6.3. Solutions were left for 5 and 24 h at rt under vigorous stirring before analysis. Same experimental conditions were applied for the preparation of fluorescently labelled PICsomes, where PEG-PGA/PEG-PGA-FITC (molar ratio 0.33:1) or PEG-PGA/PEG-PGA-Cy5 (molar ratio 1:0.08) were premixed before PIC formation.

Table S1. Concentrations of the solutions of dendrimer used in the preparation of PIC and of the final PIC assemblies. PCD refers to peripheral charge density.

Dendrimer	PCD (%)	Dendrimer concentration (mg/mL)	PIC	PIC concentration (mg/mL)
2[G3]-$(NH_2{\cdot}HCl)_{54}$	100	1.46	PIC_{100}	0.81
2[G3]-$(N_3)_8(NH_2{\cdot}HCl)_{46}$	85	1.70	PIC_{85}	0.91
2[G3]-$(N_3)_{16}(NH_2{\cdot}HCl)_{38}$	70	2.12	PIC_{70}	1.04
2[G3]-$(N_3)_{27}(NH_2{\cdot}HCl)_{27}$	50	2.86	PIC_{50}	1.35

3. Dynamic Light Scattering: Size and Stability of PIC

DLS measurements were performed on a Malvern Nano ZS (Malvern Instruments, U.K.), operating at 633 nm with a 173º scattering angle at 25 ºC. DLS mean hydrodynamic diameters (*D*) were obtained from the intensity particle size distribution provided by Malvern Zetasizer Software of 5 independent measurements. DLS histograms were obtained from the intensity particle size distributions. PIC assemblies prepared following the General Procedure III were left at 25 ºC for 5 and 24 h before DLS analysis.

Table S2. Mean hydrodynamic diameters (*D*, nm) and PDI (in brackets) of PIC assemblies prepared from 2[G3]-N_3/NH_3^+ and PEG-PGA with increasing NaCl concentration.

Peripheral Charge Density (PCD) - *Reduction Degree*		NaCl Concentration (mM) 0	75	100	125	150	175	200	225	235	250	275	300	310
100%	*5 h*	76 ± 1<br>(0.01 ± 0.01)	92 ± 1<br>(0.06 ± 0.01)	114 ± 2<br>(0.06 ± 0.01)	180 ± 1<br>(0.12 ± 0.01)	203 ± 2<br>(0.05± 0.02)	207 ± 2<br>(0.03 ± 0.02)	213 ± 3<br>(0.05 ± 0.02)	264 ± 7<br>(0.17 ± 0.01)		369 ± 8<br>(0.16 ± 0.02)	518 ± 14<br>(0.22 ± 0.01)	614 ± 29<br>(0.28 ± 0.01)	784 ± 16<br>(0.29 ± 0.01)
	24 h	**79 ± 1<br>(0.03 ± 0.01)**	**92 ± 1<br>(0.05 ± 0.01)**	**121 ± 1<br>(0.04 ± 0.01)**	**194 ± 1<br>(0.15 ± 0.01)**	**227 ± 3<br>(0.10 ± 0.01)**	**237 ± 7<br>(0.10 ± 0.03)**	**266 ± 10<br>(0.16 ± 0.02)**	**380 ± 9<br>(0.20 ± 0.01)**		**515 ± 7<br>(0.22 ± 0.01)**	**664 ± 24<br>(0.26 ± 0.01)**	**741 ± 42<br>(0.30 ± 0.02)**	**1050 ± 37<br>(0.24 ± 0.01)**
85%	*5 h*	95 ± 1<br>(0.03 ± 0.01)	166 ± 3<br>(0.10 ± 0.02)	196 ± 4<br>(0.05 ± 0.03)	200 ± 2<br>(0.10 ± 0.02)	224 ± 4<br>(0.08 ± 0.02)	296 ± 8<br>(0.17 ± 0.01)	526 ± 14<br>(0.20 ± 0.02)	670 ± 33<br>(0.27 ± 0.01)	720 ± 29<br>(0.20 ± 0.02)				
	24 h	**109 ± 1<br>(0.05 ± 0.01)**	**182 ± 2<br>(0.11 ± 0.01)**	**220 ± 3<br>(0.08 ± 0.01)**	**260 ± 7<br>(0.13 ± 0.02)**	**344 ± 3<br>(0.22 ± 0.02)**	**443 ± 8<br>(0.19 ± 0.01)**	**639 ± 14<br>(0.26 ± 0.01)**	**828 ± 55<br>(0.30 ± 0.01)**	**928 ± 20<br>(0.30 ± 0.01)**				
70%	*5 h*	103 ± 1<br>(0.03 ± 0.01)	189 ± 2<br>(0.02 ± 0.02)	195 ± 3<br>(0.06 ± 0.01)	231 ± 3<br>(0.08 ± 0.02)	272 ± 7<br>(0.15± 0.02)	451 ± 9<br>(0.16 ± 0.01)	732 ± 20<br>(0.26 ± 0.02)	825 ± 49<br>(0.26 ± 0.02)					
	24 h	**124 ± 1<br>(0.04 ± 0.02)**	**208 ± 4<br>(0.06 ± 0.02)**	**253 ± 8<br>(0.13 ± 0.01)**	**295 ± 4<br>(0.20 ± 0.02)**	**414 ± 7<br>(0.20 ± 0.01)**	**563 ± 10<br>(0.19 ± 0.01)**	**835 ± 17<br>(0.32 ± 0.04)**	**1281 ± 82<br>(0.21 ± 0.03)**					
50%	*5 h*	170 ± 1<br>(0.04 ± 0.01)	212 ± 3<br>(0.07 ± 0.04)	386 ± 7<br>(0.17 ± 0.02)	649 ± 22<br>(0.21 ± 0.02)	769 ± 19<br>(0.29 ± 0.02)								
	24 h	**181 ± 1<br>(0.02 ± 0.01)**	**240 ± 4<br>(0.08 ± 0.02)**	**475 ± 23<br>(0.19 ± 0.02)**	**753 ± 18<br>(0.27 ± 0.01)**	**1205 ± 48<br>(0.16 ± 0.04)**								

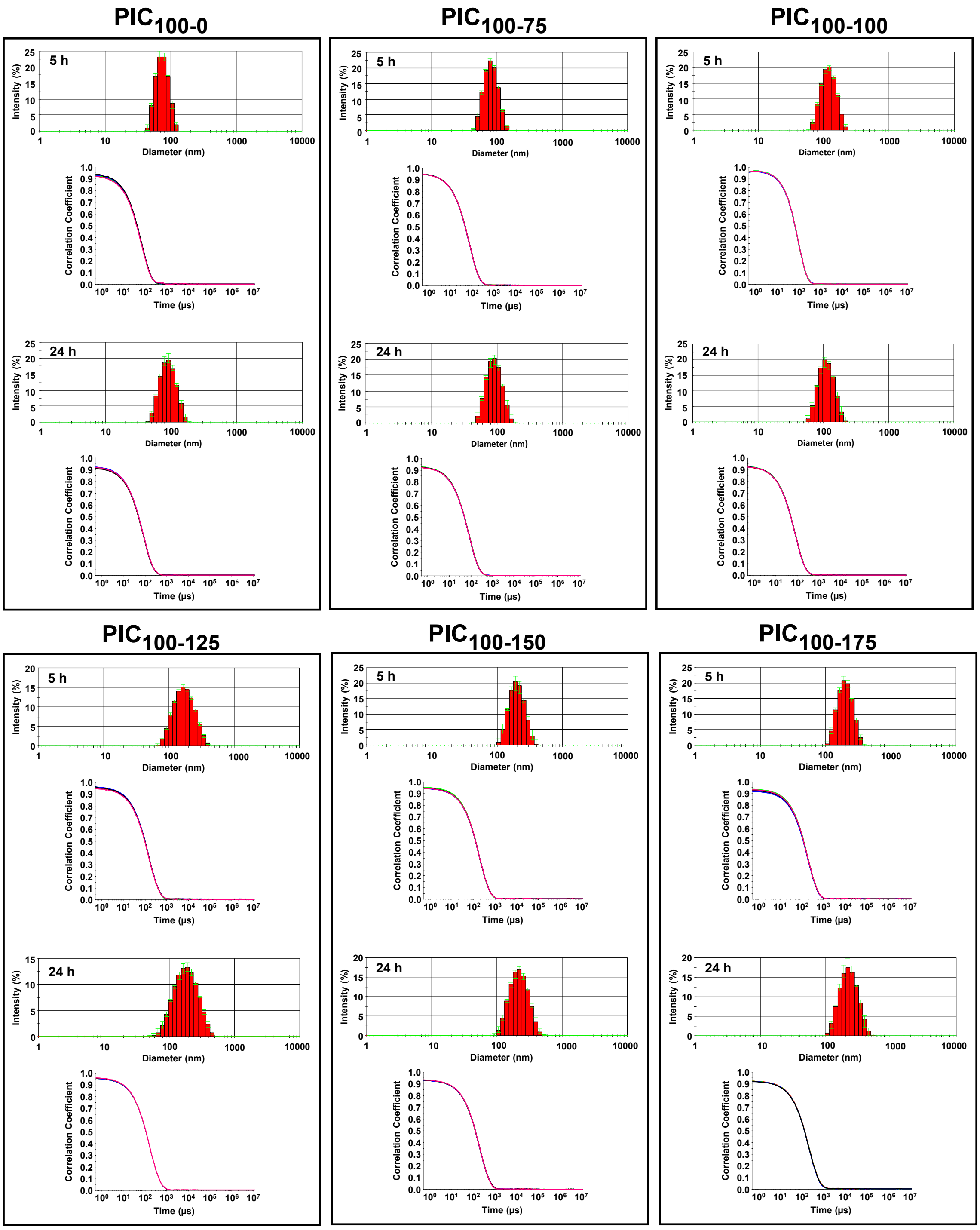

Figure S1. DLS histograms and correlation functions (5 and 24 h) of PIC_{100} assemblies prepared from 2[G3]-$(NH_2{\cdot}HCl)_{54}$ and PEG-PGA in 10 mM PB pH 6.2 supplemented with 0-175 mM NaCl.

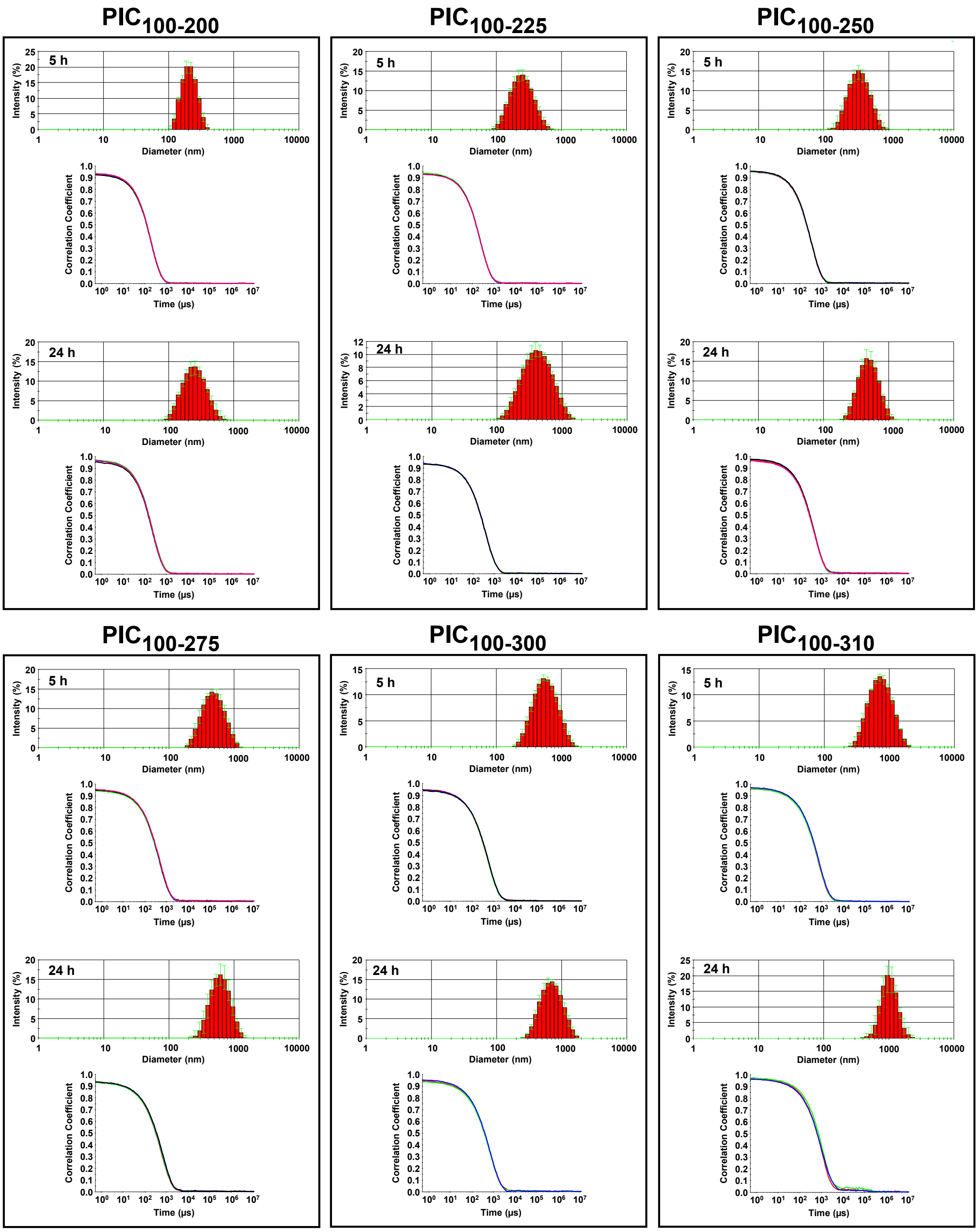

Figure S2. DLS histograms and correlation functions (5 and 24 h) of PIC$_{100}$ assemblies prepared from 2[G3]-$(NH_2{\cdot}HCl)_{54}$ PEG-PGA and in 10 mM PB pH 6.2 supplemented with 200-310 mM NaCl.

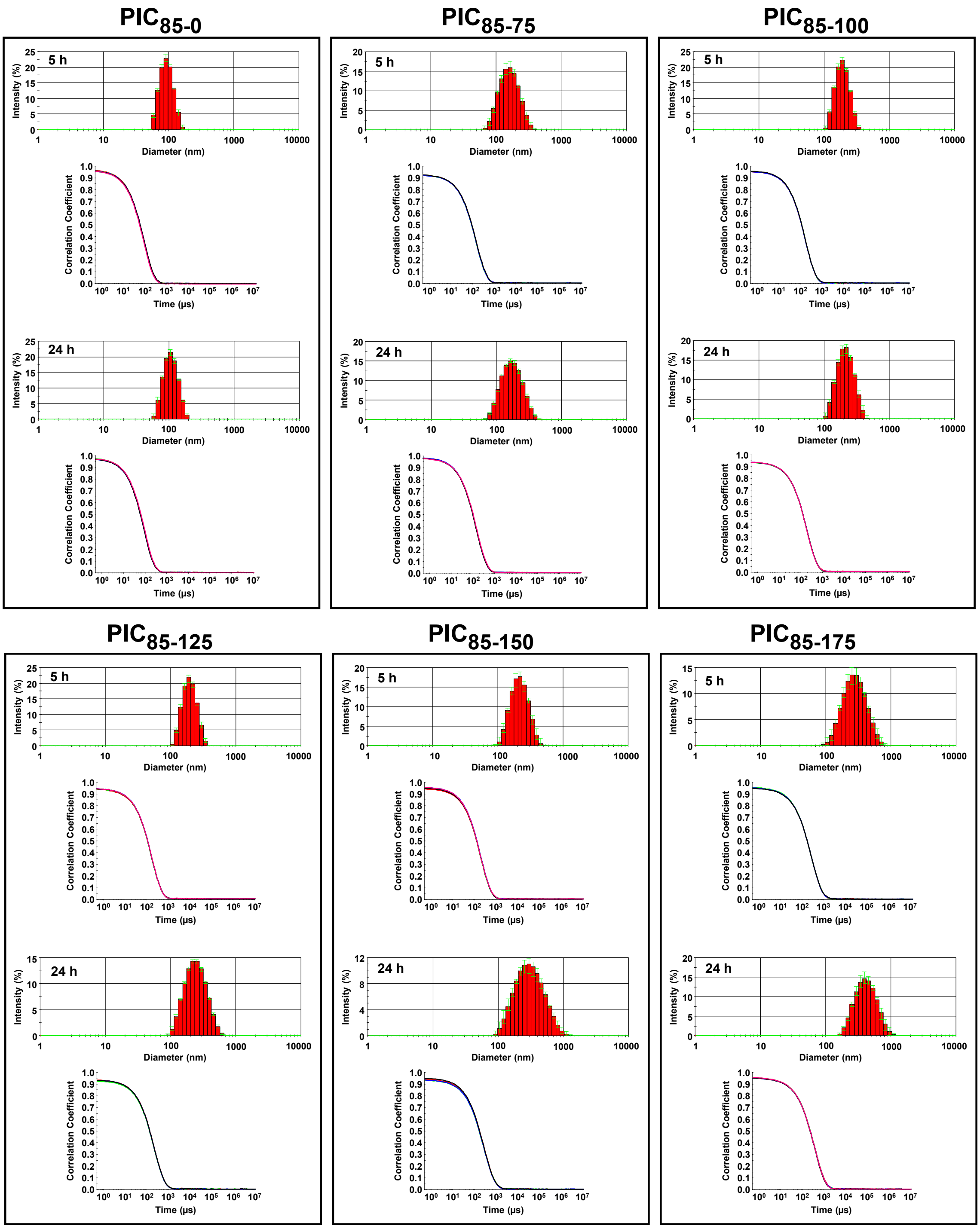

Figure S3. DLS histograms and correlation functions (5 and 24 h) of PIC_{85} assemblies prepared from 2[G3]-$(N_3)_8(NH_2{\cdot}HCl)_{46}$ and PEG-PGA in 10 mM PB pH 6.2 supplemented with 0-175 mM NaCl.

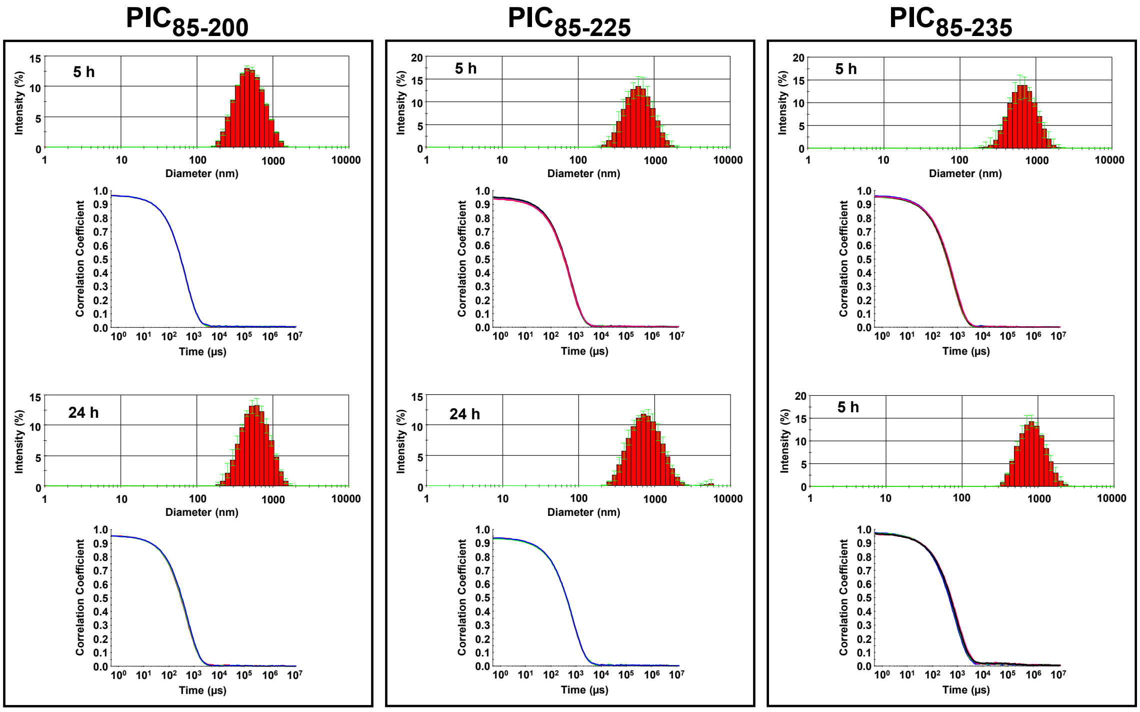

Figure S4. DLS histograms and correlation functions (5 and 24 h) of PIC_{85} assemblies prepared from 2[G3]-$(N_3)_8(NH_2{\cdot}HCl)_{46}$ and PEG-PGA in 10 mM PB pH 6.2 supplemented with 200-235 mM NaCl.

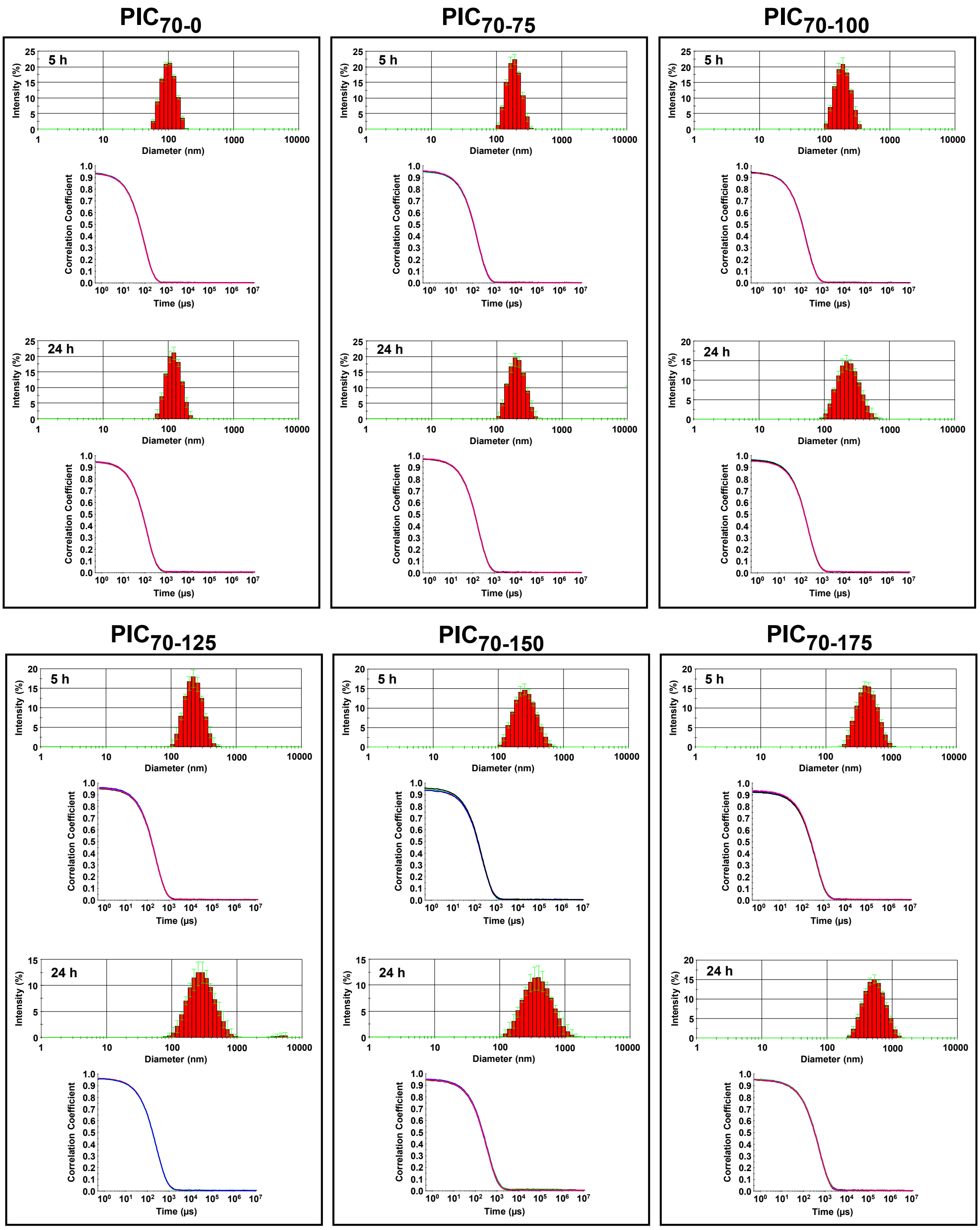

Figure S5. DLS histograms and correlation functions (5 and 24 h) of PIC_{70} assemblies prepared from $2[G3]\text{-}(N_3)_{16}(NH_2\cdot HCl)_{38}$ and PEG-PGA in 10 mM PB pH 6.2 supplemented with 0-175 mM NaCl.

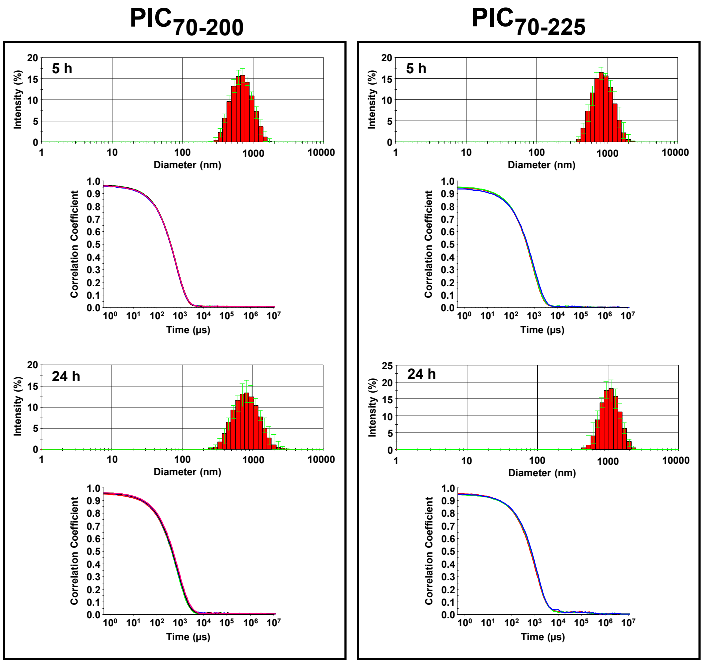

Figure S6. DLS histograms and correlation functions (5 and 24 h) of PIC_{70} assemblies prepared from 2[G3]-$(N_3)_{16}(NH_2{\cdot}HCl)_{38}$ and PEG-PGA in 10 mM PB pH 6.2 with supplemented 200-225 mM NaCl.

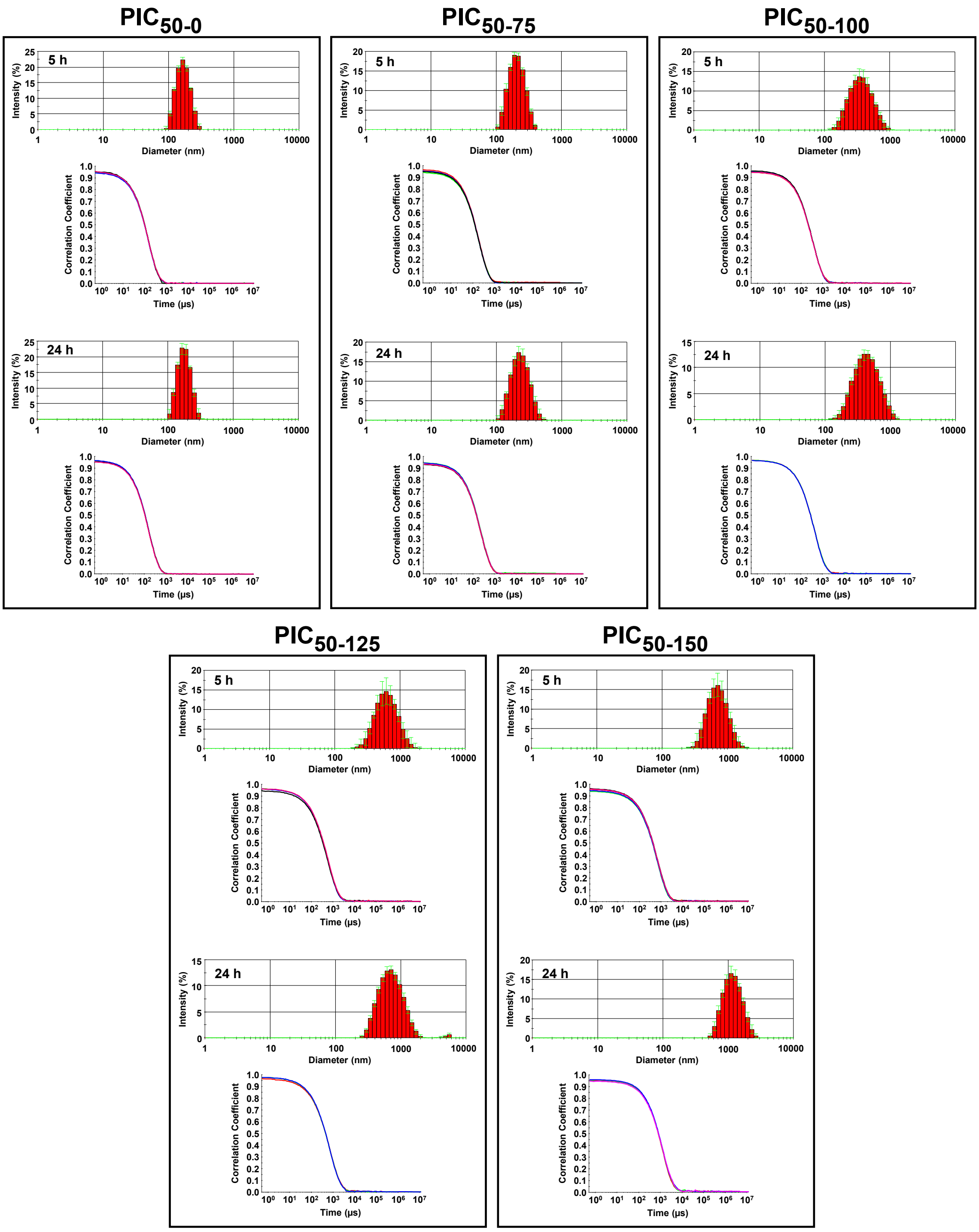

Figure S7. DLS histograms and correlation functions (5 and 24 h) of PIC_{50} assemblies prepared from 2[G3]-$(N_3)_{27}(NH_2{\cdot}HCl)_{27}$ and PEG-PGA in 10 mM PB pH 6.2 supplemented with 0-150 mM NaCl.

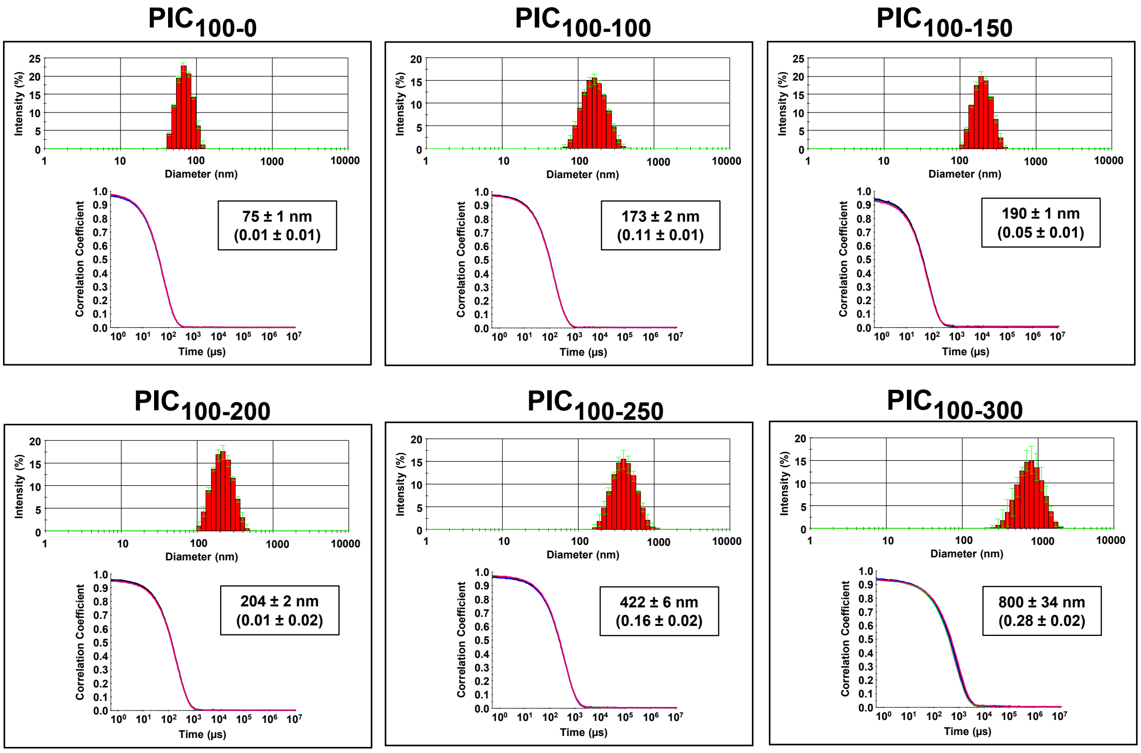

Figure S8. DLS histograms and correlation functions of PIC_{100-0} after 1 h of preparation in 10 mM PB pH 6.2 and after 7 h of subsequent dialysis against 10 mM PB pH 6.2 supplemented with 100-300 mM NaCl. Mean hydrodynamic diameters (nm) and PDI (in brackets).

4. Fitting the Size Variation of PIC with the NaCl Concentration and the Peripheral Charge Density (PCD) of the Dendrimer: Derivation of Eq 4

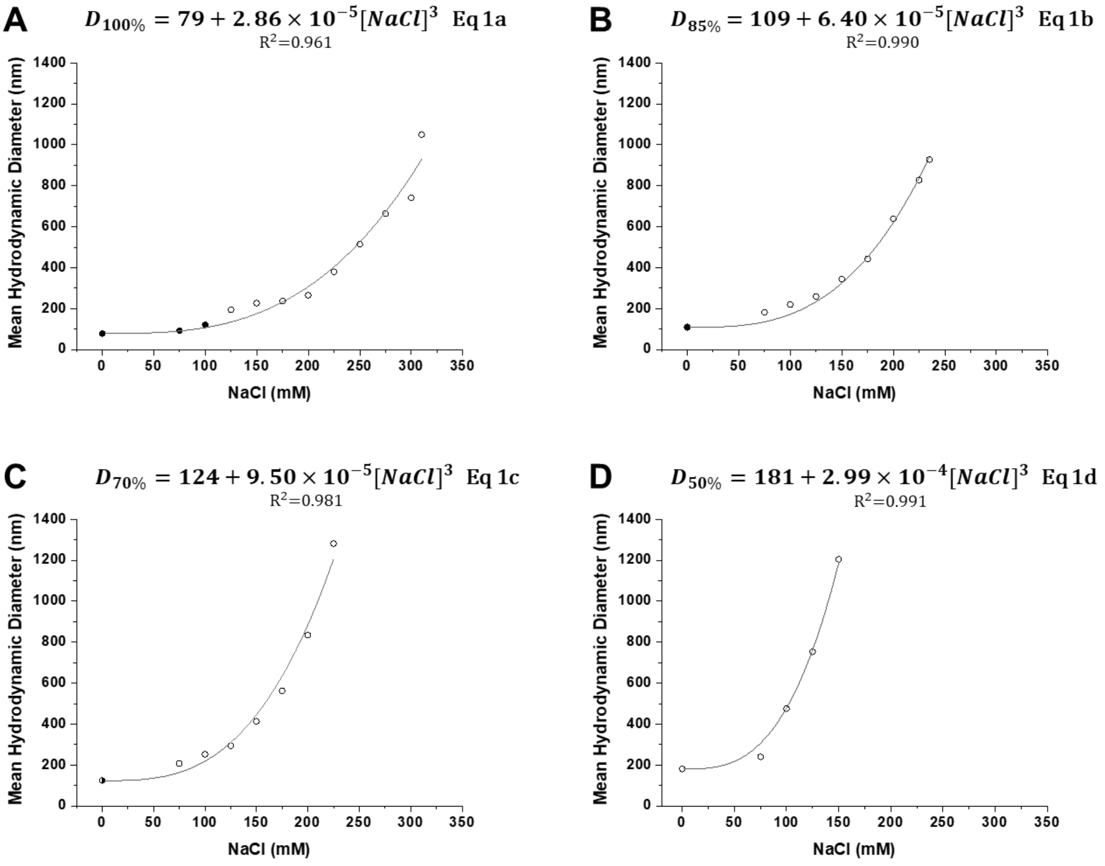

$$D = a + b\,[NaCl]^3 \text{ (Eq 1)}$$

Figure S9. Variation of the mean hydrodynamic diameter (*D*) of PIC assemblies with the NaCl concentration ([NaCl], mM) as a function of the peripheral charge density (PCD) of the dendrimer: 100% (**A**), 85% (**B**), 70% (**C**), and 50% (**D**). Each plot was fitted to a cubic power function (Eqs 1a-d), where the intercepts equal the *D* values obtained in the absence of NaCl. R^2 (R-squared) is the coefficient of determination. Fittings were done using Origin 8.5 software. Filled circles refer to micelles and open circles to vesicles.

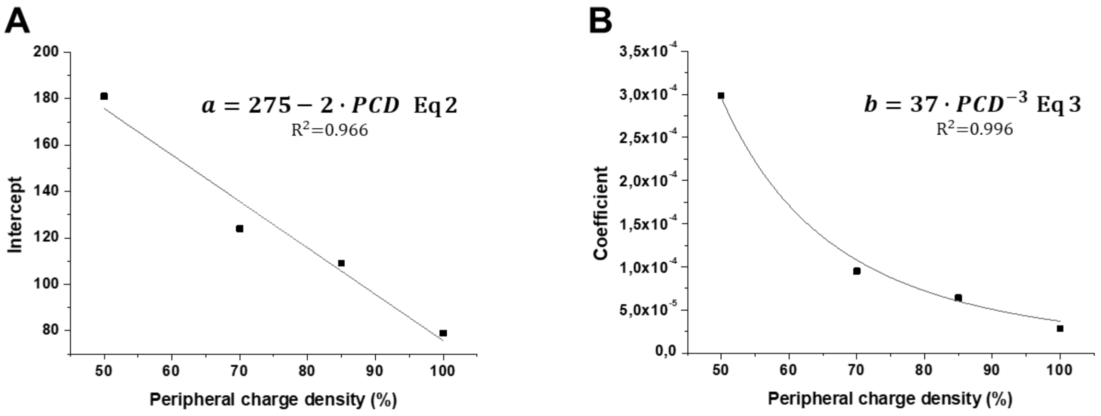

$$D = 275 - 2 \cdot PCD + \frac{37}{PCD^3}[NaCl]^3 \quad \textbf{Eq 4}$$

R^2=0.923

Figure S10. The plots of the variations of the intercepts and coefficients in Eq 1a-d versus the peripheral charge density (PCD) of the dendrimer fit a straight line (Eq 2, **A**) and the inverse of a cubic power function (Eq 3, **B**), respectively. R^2 (R-squared) is the coefficient of determination. Fittings were done using Origin 8.5 software. Substituting Eqs 2 and 3 into Eq 1 afforded Eq 4, which allows predicting the hydrodynamic diameter (*D*) of PIC assemblies by simply selecting the PCD of the dendrimer and NaCl concentration of the medium.

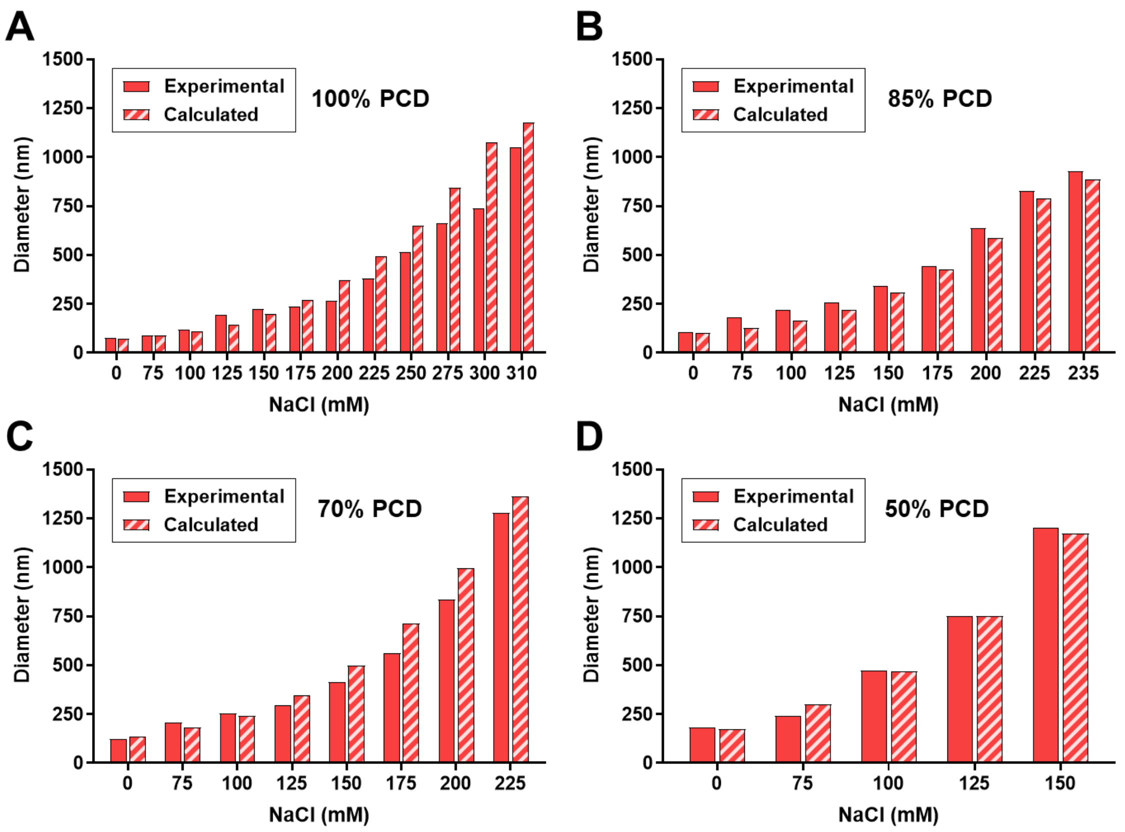

Figure S11. Experimental mean hydrodynamic diameters and calculated values using Eq 4 for PIC assemblies prepared from dendrimers with different peripheral charge density (PCD) at different NaCl concentrations (**A**, 100% PCD; **B**, 85% PCD; **C**, 70% PCD; **D**, 50% PCD).

5. Measurement of Z-Potential

PIC assemblies prepared following the General Procedure III (5 h) were crosslinked overnight with EDC (5 equiv per amino group, added from a fresh 100 mg/mL solution in H_2O) and then dialyzed (MWCO 1 kDa) for 1 h against H_2O (3 × 200 mL). Z-potential values were obtained by laser doppler anemometry (LDA), measuring the mean electrophoretic mobility (Malvern Zetasizer Nano ZS, Malvern Instruments). Measurements were performed in H_2O supplemented with 10 mM NaCl (Smoluchowski approximation). Z-potential values close to zero were obtained: $PIC_{100\text{-}0}$ (-0.24 mV), $PIC_{100\text{-}150}$ (0.81 mV), $PIC_{100\text{-}300}$ (-0.39 mV).

6. Cryo-Transmission Electron Microscopy (cryo-TEM)

For 2D cryo-imaging, 3 µL of PIC assemblies (0.5 mg/mL) prepared following the General Procedure III were directly applied onto a glow-discharged 300-mesh Quantifoil Cu/Rh R 2/2 holey carbon grid and rapidly vitrified by plunging into liquid ethane using a ThermoFisher Scientific Vitrobot Mark IV. The vitrified samples were then transferred into a Talos Arctica (ThermoFisher Scientific) operating at liquid nitrogen temperature (200 kV). Images were acquired under low-dose conditions using a Falcon 3EC Direct Electron Detector (ThermoFisher Scientific), with a nominal magnification between 13500× and 73000× and defocus range of -1.5 to -3.0 µm. Average diameters of PIC assemblies and thickness of PICsome membranes were determined with ImageJ software (version 1.51j8) measuring the line intensity profile across a representative number of assemblies (Figure S12) and PICsome membranes.

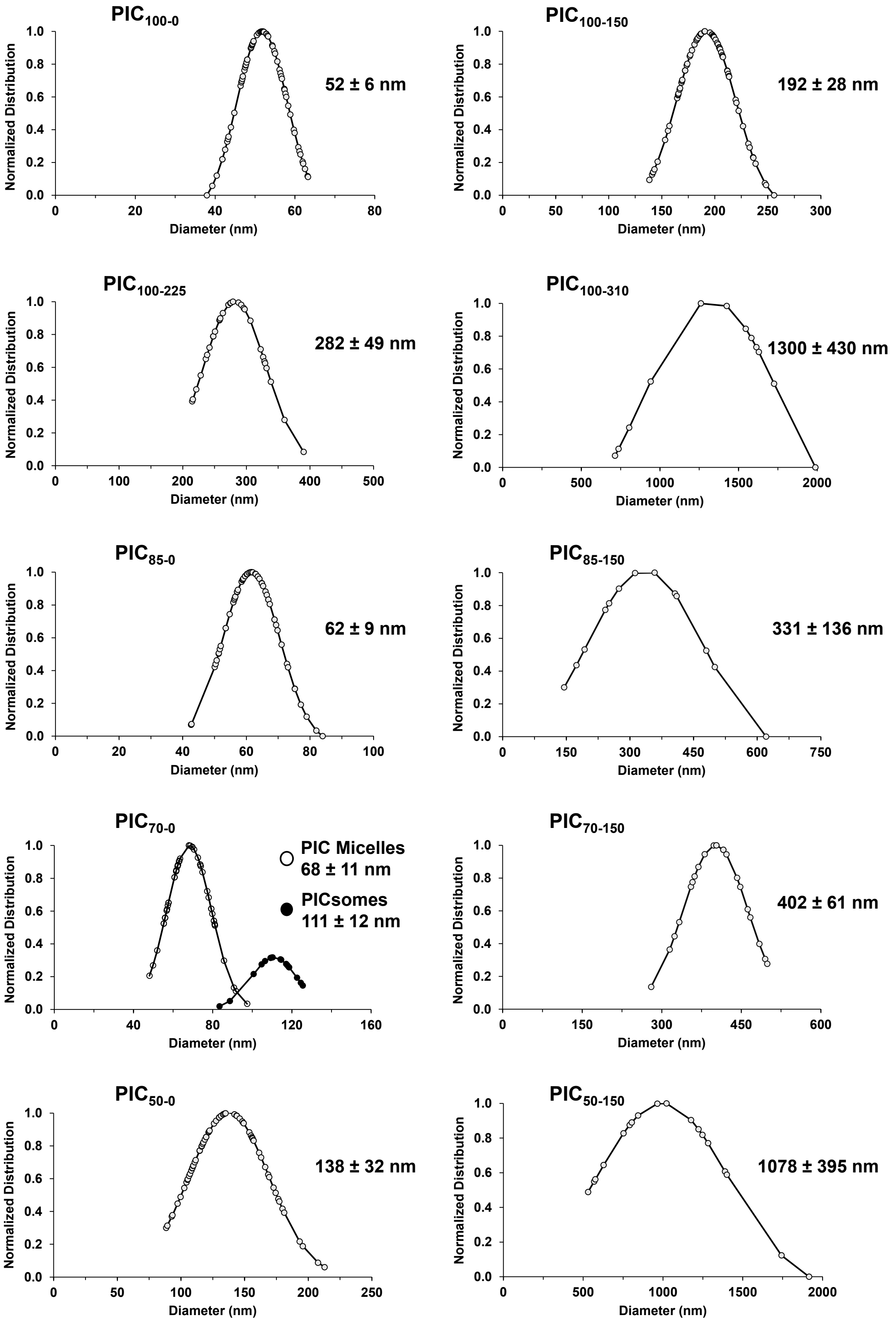

Figure S12. Normalized statistical size distribution of PIC assemblies by cryo-TEM determined measuring the line intensity profile with ImageJ software (version 1.51j8).

7. Encapsulation of Enzymes

PICsomes were crosslinked with EDC to prevent disassembly upon contact with the well during confocal microscopy analysis. No enzyme charges were considered for the charge-neutralized PIC formation.

***HRP-Cy5@PIC$_{100\text{-}300}$*.** HRP-Cy5 (47 µL, 2 mg/mL) in 10 mM PB pH 6.2, 300 mM NaCl was added to a solution of 2[G3]-(NH_2·HCl)$_{54}$ (300 µL, 1.46 mg/mL) in 10 mM NaH_2PO_4/0.1 M HCl (10% v/v), 300 mM NaCl. After 2 min, PEG-PGA (600 µL, 0.5 mg/mL) in 10 mM Na_2HPO_4, 300 mM NaCl was added, and the mixture was stirred protected from light. After 4 h, HRP-Cy5@PIC$_{100\text{-}300}$ was crosslinked overnight with EDC (10 equiv per amino group, added from a fresh 100 mg/mL solution in H_2O) and dialyzed (MWCO 1000 kDa) for 6 h against 10 mM PB pH 7.4, 150 mM NaCl (3 × 200 mL) to remove unencapsulated protein. Complete removal of free HRP-Cy5 was confirmed by SDS-PAGE (8%).

***GOX-AF488@PIC$_{100\text{-}300}$*.** GOX-AF488 (176 µL, 2 mg/mL) in 10 mM PB pH 6.2, 300 mM NaCl was added to a solution of PEG-PGA (600 µL, 0.5 mg/mL) in 10 mM Na_2HPO_4, 300 mM NaCl. After 2 min, the mixture was added to a solution of 2[G3]-(NH_2·HCl)$_{54}$ (300 µL, 1.46 mg/mL) in 10 mM NaH_2PO_4/0.1 M HCl (10% v/v), 300 mM NaCl. After 4 h of stirring protected from light, GOX-AF488@PIC$_{100\text{-}300}$ was crosslinked overnight with EDC (10 equiv per amino group, added from a fresh 100 mg/mL solution in H_2O) and dialyzed (MWCO 1000 kDa) for 6 h against 10 mM PB pH 7.4, 150 mM NaCl (3 × 200 mL) to remove unencapsulated protein. Complete removal of free GOX-AF488 was confirmed by SDS-PAGE (8%).

Encapsulation efficiency of enzymes. The encapsulation efficiency of enzymes (defined as the fraction of loaded enzyme relative to the total amount of added enzyme) was determined by SDS-PAGE using non-crosslinked PICsomes. After protein (HRP-Cy5 or GOX-AF488) loading, free enzyme was removed by size exclusion chromatography (PD-10 column, Sephadex G-25, 1 M NaCl) and quantified by SDS-PAGE (12%, staining with Coomassie Brilliant Blue R-250 or fluorescence) by comparison with a standard calibration curve made with fresh enzyme solutions of known concentrations. An encapsulation efficiency of 85 ± 4% was determined for GOX and 62 ± 8% for HRP. These values account for enzyme loadings (defined as the mass fraction of loaded enzyme relative to enzyme-loaded PICsome) of 28% for GOX and 7.2% for HRP.

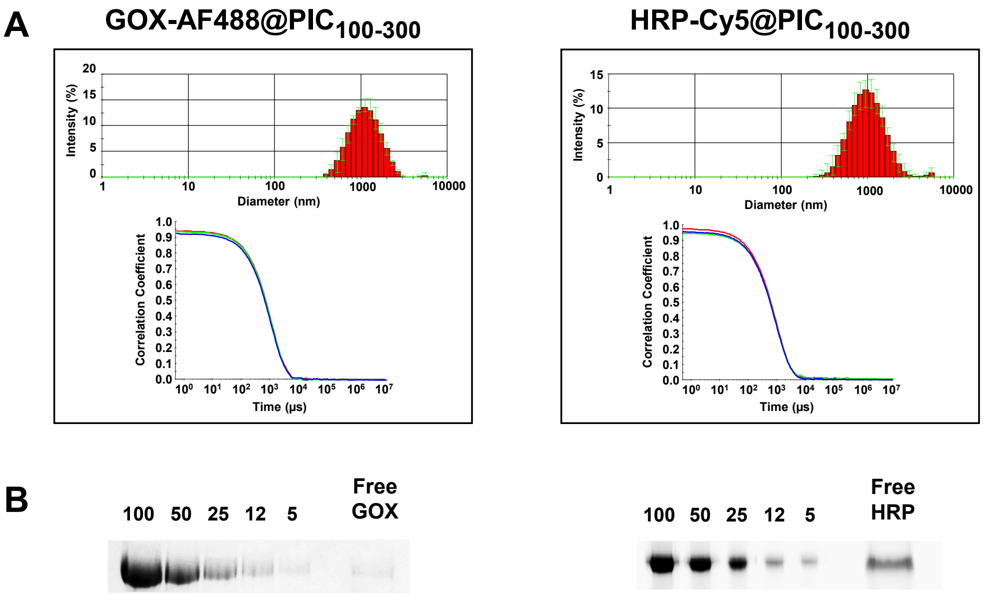

Figure S13. DLS histograms and correlation functions of GOX-AF488@PIC$_{100\text{-}300}$ and HRP-Cy5@PIC$_{100\text{-}300}$ in 10 mM PB pH 7.4, 150 mM NaCl (**A**). Determination of the encapsulation efficiency of the enzymes by SDS-PAGE (**B**).

8. Enzymatic Cascade Assays

Monitoring of the enzymatic cascade reaction by CLSM (Amplex Red). GOX-AF488@PIC$_{100-300}$ and HRP-Cy5@PIC$_{100-300}$ in 10 mM PB pH 7.4, 150 mM NaCl were mixed in a 1:1 ratio to a volume of 150 µL in a microscope slide (Cellvis 4-Chamber microwells 35 mm Glass Bottom Dish with 20 mm, #1.5 cover glass). An image was acquired at t = 0 after the addition of Amplex Red (5 µL, 6 µM in DMSO) to record the starting amount of resorufin present at background levels in Amplex Red. The enzymatic cascade reaction was monitored after addition of glucose (5 µL, 120 µM in 10 mM PB pH 7.4, 150 mM NaCl) taking images at different time points. Control experiments were also performed under identical conditions in assays without GOX-AF488@PIC$_{100-300}$ or HRP-Cy5@PIC$_{100-300}$.

The individual channels were recorded sequentially using the following parameters:

- Red channel (resorufin): excitation at 561 nm; emission at 620/50 nm.
- Green channel (GOX): excitation at 488 nm; emission at 525/50 nm.
- Blue channel (HRP): excitation at 637 nm; emission at 725/40 nm.

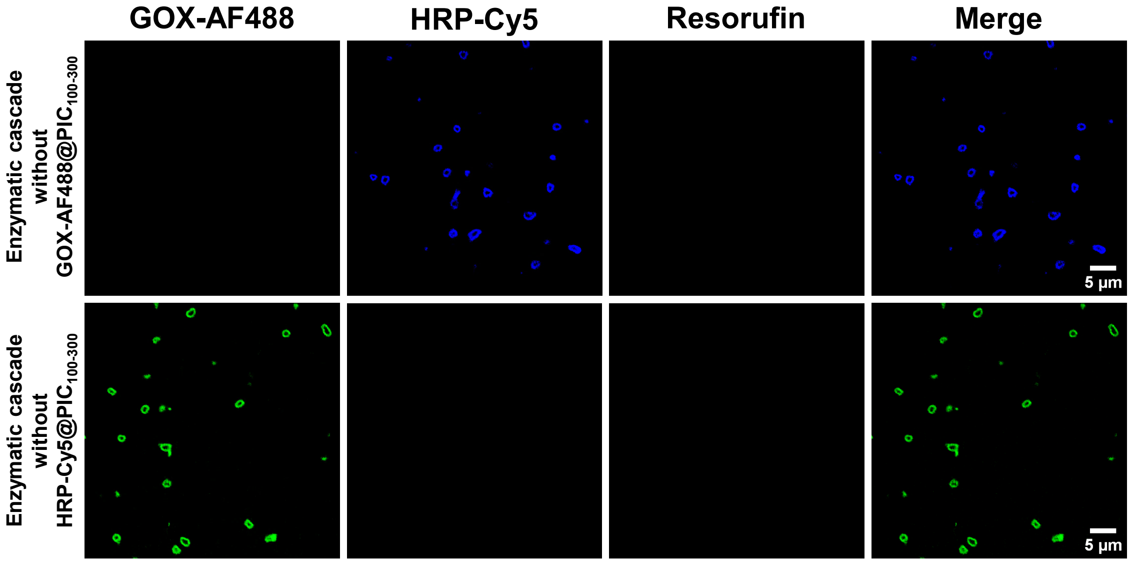

Figure S14. CLSM images (5 min) of control enzymatic cascade experiments performed without GOX-AF488@PIC$_{100-300}$ (top) or HRP-Cy5@PIC$_{100-300}$ (bottom).

Monitoring of the enzymatic cascade reaction by absorbance (ABTS). GOX@PIC$_{100-300}$ and HRP@PIC$_{100-300}$ in 10 mM PB pH 7.4, 150 mM NaCl were mixed in a 1:1 ratio to a volume of 150 µL in a 96-well plate. ABTS (5 µL, 3.20 mM in 10 mM PB pH 7.4, 150 mM NaCl) and glucose (5 µL, 160 mM in 10 mM PB pH 7.4, 150 mM NaCl) were added. The reaction progress (0.93 µM GOX and 0.79 µM HRP) was monitored by measuring the absorbance of the ABTS radical cation (405 nm) in a plate reader (Tecan Infinite F200 pro). Control experiments performed under identical conditions in assays without glucose, GOX@PIC$_{100-300}$, or HRP@PIC$_{100-300}$ resulted in zero increase in absorbance in all cases.

Protection against Proteinase K. GOX@PIC$_{100-300}$ and HRP@PIC$_{100-300}$ in 10 mM PB pH 7.4, 150 mM NaCl were mixed in a 1:1 ratio to a volume of 1.2 mL (equivalent to 0.93 µM GOX and 0.79 µM HRP). Half of this solution was incubated in the absence of proteinase K (control) and the other half in the presence of 10 µM proteinase K (pK; 10 µL, 0.6 mM in 10 mM PB pH 7.4, 150 mM NaCl) for 24 h at 37 ºC. Then, the mixture containing pK was dialyzed (MWCO 1000 kDa; 10 mM PB pH 7.4, 150 mM NaCl) to remove pK.

The protection conferred by the PICsome on GOX and HRP against pK was assessed by comparing the enzymatic activity of the PICsomes incubated with pK to that of the control PICsomes. To this end, 150 µL of each PICsome mixture were transferred to a 96-well plate. ABTS (5 µL, 3.2 mM in 10 mM PB pH 7.4, 150 mM NaCl) and glucose (5 µL, 160 mM in 10 mM PB pH 7.4, 150 mM NaCl) were added and the production of the ABTS radical cation was monitored by measuring the absorbance at 405 nm in a plate reader (Tecan Infinite F200 pro). While the PICsomes incubated with pK retained 86 ± 2% of the enzymatic activity of the control PICsomes, a solution of free GOX and HRP (0.93

μM GOX, 0.79 μM HRP in 10 mM PB pH 7.4, 150 mM NaCl) incubated for 24 h at 37 ºC with 10 μM pK showed null enzymatic activity.

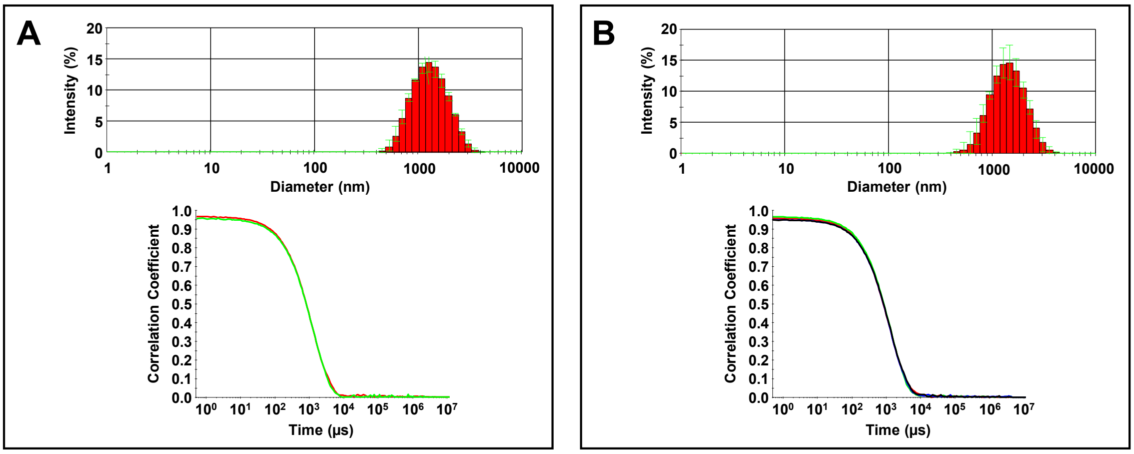

Figure S15. DLS histograms and correlation functions of a mixture of GOX@PIC100-300 and HRP@PICs100-300 in 10 mM PB pH 7.4, 150 mM NaCl after incubation at 37 °C for 24 h in the absence (**A**) and the presence of 10 μM pK (**B**).

9. Stability in Cell Culture Medium

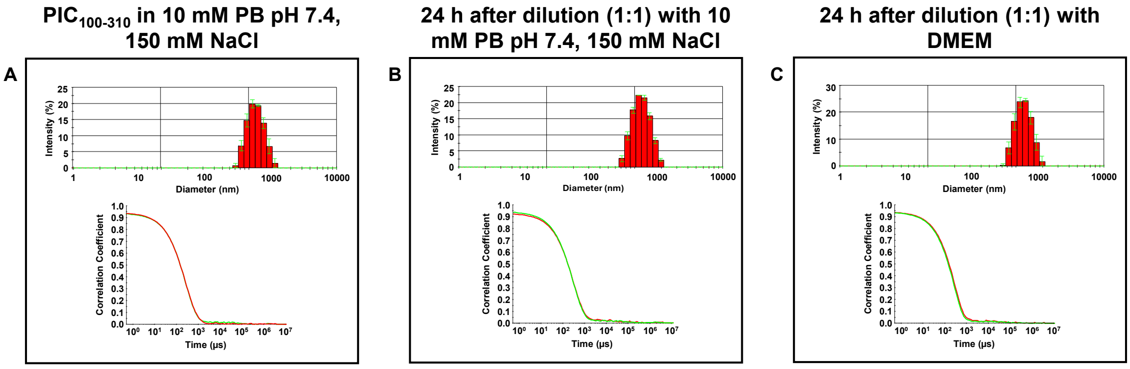

Figure S16. DLS histograms and correlation functions of PIC100-310 before (**A**) and after 24 h of dilution (1:1) with 10 mM PB pH 7.4, 150 mM NaCl (**B**) or Dulbecco's modified Eagle's medium (DMEM) with high glucose (**C**).

10. References

1. Amaral, S. P.; Fernandez-Villamarin, M.; Correa, J.; Riguera, R.; Fernandez-Megia, E. Efficient Multigram Synthesis of the Repeating Unit of Gallic Acid-Triethylene Glycol Dendrimers. *Org. Lett.* **2011**, *13*, 4522-4525.

2. Bräse, S.; Gil, C.; Knepper, K.; Zimmermann, V. Organic Azides: An Exploding Diversity of a Unique Class of Compounds. *Angew. Chem., Int. Ed.* **2005**, *44*, 5188-5240. and references therein.

3. Kolb, H. C.; Finn, M. G.; Sharpless, K. B. Click Chemistry: Diverse Chemical Function from a Few Good Reactions. *Angew. Chem., Int. Ed.* **2001**, *40*, 2004-2021. and references therein.
